# Transplantation of mesenchymal stem cells ameliorates secondary osteoporosis through interleukin-17-impaired functions of recipient bone marrow mesenchymal stem cells in MRL/*lpr* mice

**DOI:** 10.1186/s13287-015-0091-4

**Published:** 2015-05-27

**Authors:** Lan Ma, Reona Aijima, Yoshihiro Hoshino, Haruyoshi Yamaza, Erika Tomoda, Yosuke Tanaka, Soichiro Sonoda, Guangtai Song, Wei Zhao, Kazuaki Nonaka, Songtao Shi, Takayoshi Yamaza

**Affiliations:** Department of Molecular Cell Biology and Oral Anatomy, Kyushu University Graduate School of Dental Science, 3-1-1 Maidashi, Higashi-ku, Fukuoka 812-8582 Japan; Department of Pediatric Dentistry, Kyushu University Graduate School of Dental Science, 3-1-1 Maidashi, Higashi-ku, Fukuoka 812-8582 Japan; Department of Pediatric Dentistry, Guanghua School of Stomatology, Hospital of Stomatology, Sun Yat-sen University, No. 56, Lingyuan Xi Road, Guangzhou, 510055 China; Department of Pediatric Dentistry, School of Stomatology, Wuhan University, Luo-jia-shan, Wuchang, Wuhan, 430072 China; Guangdong Provincial Key Laboratory of Stomatology, Sun Yat-sen University, No. 135, Xingang Xi Road, Guangzhou, 510275 China; Center for Craniofacial Molecular Biology, Herman Ostrow School of Dentistry of USC, University of Southern California, 2250 Alcazar Street, Los Angeles, CA 90033-9062 USA; Department of Anatomy and Cell Biology, School of Dental Medicine, University of Pennsylvania, 240 South 40th Street, Philadelphia, PA 19204-6030 USA

## Abstract

**Introduction:**

Secondary osteoporosis is common in systemic lupus erythematosus and leads to a reduction in quality of life due to fragility fractures, even in patients with improvement of the primary disorder. Systemic transplantation of mesenchymal stem cells could ameliorate bone loss and autoimmune disorders in a MRL/*lpr* mouse systemic lupus erythematosus model, but the detailed therapeutic mechanism of bone regeneration is not fully understood. In this study, we transplanted human bone marrow mesenchymal stem cells (BMMSCs) and stem cells from exfoliated deciduous teeth (SHED) into MRL/*lpr* mice and explored their therapeutic mechanisms in secondary osteoporotic disorders of the systemic lupus erythematosus model mice.

**Methods:**

The effects of systemic human mesenchymal stem cell transplantation on bone loss of MRL*/lpr* mice were analyzed in vivo and ex vivo. After systemic human mesenchymal stem cell transplantation, recipient BMMSC functions of MRL*/lpr* mice were assessed for aspects of stemness, osteogenesis and osteoclastogenesis, and a series of co-culture experiments under osteogenic or osteoclastogenic inductions were performed to examine the efficacy of interleukin (IL)-17-impaired recipient BMMSCs in the bone marrow of MRL*/lpr* mice.

**Results:**

Systemic transplantation of human BMMSCs and SHED recovered the reduction in bone density and structure in MRL/*lpr* mice. To explore the mechanism, we found that impaired recipient BMMSCs mediated the negative bone metabolic turnover by enhanced osteoclastogenesis and suppressed osteoblastogenesis in secondary osteoporosis of MRL/*lpr* mice. Moreover, IL-17-dependent hyperimmune conditions in the recipient bone marrow of MRL/*lpr* mice damaged recipient BMMSCs to suppress osteoblast capacity and accelerate osteoclast induction. To overcome the abnormal bone metabolism, systemic transplantation of human BMMSCs and SHED into MRL/*lpr* mice improved the functionally impaired recipient BMMSCs through IL-17 suppression in the recipient bone marrow and then maintained a regular positive bone metabolism via the balance of osteoblasts and osteoclasts.

**Conclusions:**

These findings indicate that IL-17 and recipient BMMSCs might be a therapeutic target for secondary osteoporosis in systemic lupus erythematosus.

**Electronic supplementary material:**

The online version of this article (doi:10.1186/s13287-015-0091-4) contains supplementary material, which is available to authorized users.

## Introduction

Osteoporosis is defined as a reduction in bone strength and is the most common bone disease [1]. The bone loss is primarily related to age and/or menopause and secondarily affected by underlying risk factors such as nutritional deficiencies, diseases, or drugs [2]. Systemic lupus erythematosus (SLE) is a refractory and chronic multiorgan autoimmune disease. Because recent medical advances have successfully increased the lifespan of patients with SLE, many clinical researchers have focused on the organ damage associated with the systemic chronic inflammation and/or long-term medications relating to quality of life [3]. Secondary osteoporosis frequently occurs in SLE patients, which causes fragility fractures [4]. Currently, there are no safe or efficient treatments for SLE-associated osteoporosis.

Mesenchymal stem cells (MSCs) are a typical type of adult stem cell with the capabilities of self-renewal and multilineage differentiation [5]. Recent studies show that MSCs have immunomodulatory effects on immune cells [6, 7], and MSC-based cell therapy has been greatly focused on the treatment of various immune diseases such as acute graft-versus-host disease [8] and inflammatory bowel disease [9]. Previous allogeneic transplantation of human bone marrow MSCs (hBMMSCs) and human umbilical cord-derived MSCs (hUCMSCs) governs successful therapeutic efficacy in refractory SLE patients [10–12]. However, it is unclear whether MSC transplantation is an effective treatment for skeletal disorders in SLE patients.

MRL*/lpr* mice are a well-known model of human SLE-like disorders with clinical manifestations including a short lifespan, abundant autoantibodies, glomerulonephritis, and a breakdown of self-tolerance [13]. Furthermore, MRL/*lpr* mice exhibit a severe reduction of the trabecular bone, which is associated with excessive osteoclastic bone resorption and limited osteoblastic bone formation [10]. Recent studies show that systemic transplantation of human MSCs, including hBMMSCs, hUCMSCs, stem cells from human exfoliated deciduous teeth (SHED), and human supernumerary tooth-derived stem cells, improves primary autoimmune disorders in MRL/*lpr* mice, such as elevated autoimmune antibodies, renal dysfunction, and abnormal immunity [14–18]. In addition, hBMMSC and SHED transplantation markedly recovers the bone loss in MRL/*lpr* mice [16, 17]. These results indicate that MSC transplantation might be a therapeutic approach for SLE patients who suffer from secondary osteoporosis. However, little is known about the human MSC-mediated therapeutic mechanism in the skeletal disorder of MRL/*lpr* mice.

Osteoporosis is characterized by a disruption of the balance between the formation and resorption of bone, which is associated with abnormal development of osteoclasts and osteoblasts. Increasing evidence has shown that BMMSCs from SLE patients and SLE model MRL/*lpr* mice exhibit a reduction in their bone-forming capacity both in vitro and in vivo [10, 19]. Therefore, the osteogenic deficiency of recipient BMMSCs might explain the origin of osteoporosis in SLE. Accordingly, the impaired BMMSCs might be a therapeutic target for osteoporosis. However, little is known about the processes through which recipient BMMSCs are damaged functionally or the underlying mechanism of human MSC transplantation in restoration of the reduced bone formation via recipient BMMSCs in the bone marrow under SLE conditions.

In this study, we used MRL/*lpr* mice to examine the therapeutic efficacy and mechanisms of systemically transplanted hBMMSCs and SHED in the secondary osteoporotic disorders of SLE. Moreover, we focused on the pathological and clinical contributions of recipient BMMSCs to the dysregulation of bone metabolism through osteoblasts and osteoclasts in the inflammatory bone disorder of SLE.

## Materials and methods

### Human subjects

Human exfoliated deciduous teeth were obtained as clinically discarded biological samples from five patients (5–7 years old) at the Department of Pediatric Dentistry of Kyushu University Hospital under the approval of the Kyushu University Institutional Review Board for Human Genome/Gene Research (protocol number: 393–01). Written informed consent was obtained from all parents on behalf of the participants.

### Mice

C57BL/6J-*lpr/lpr* mice (female, 8 weeks old), and pregnant and young adult C57BL/6J mice (female, 8 weeks old) were purchased from Japan SLC (Shizuoka, Japan) and CLEA Japan (Tokyo, Japan), respectively. All animal experiments were approved by the Institutional Animal Care and Use Committee of Kyushu University (protocol number: A21-044-1).

### Human MSC isolation and culture

Human MSCs were isolated based on the adherent colony-forming unit fibroblasts (CFU-F) method [20]. Bone marrow cells (BMCs) (AllCells, Berkeley, CA, USA) were seeded at 1×10^7^ cells per T-75 culture flask as described previously [21]. The dental pulp tissues of human exfoliated deciduous teeth were minced and then digested with 0.3 % collagenase type I (Worthington Biochemicals, Lakewood, NJ, USA) and 0.4 % dispase II (Sanko Junyaku, Tokyo, Japan) for 60 minutes at 37 °C as reported previously [16]. Single cell suspensions were obtained by passing the digested tissue through a 70-μm cell strainer (BD Bioscience, San Jones, CA, USA) and then seeded at 1×10^6^ cells per T-75 culture flask. After 3 h, non-adherent cells were removed by washing with phosphate-buffered saline (PBS). Adherent cells were cultured at 37 °C in 5 % CO_2_. The growth medium consisted of α-minimum essential medium (αMEM; Invitrogen, Carlsbad, CA, USA) supplemented with 15 % fetal bovine serum (FBS; Equitech-Bio, Kerrville, TX, USA), 100 μM L-ascorbic acid 2-phosphate (Wako Pure Chemical Industrials, Osaka, Japan), 2 mM L-glutamine (Nacalai Tesque, Kyoto, Japan), and mixed antibiotics containing 100 U/ml penicillin and 100 μg/ml streptomycin (Nacalai Tesque). The growth medium was changed twice a week. Adherent colony-forming cells were subcultured after 14–16 days.

To identify the isolated cells as MSCs, passage 3 (P3) cells (1×10^5^/100 μl) were stained with specific antibodies against stem cell markers including CD11b, CD14, CD35, CD45, CD73, CD90, CD105 and CD146 (1 μg/ml each; eBioscience, San Diego, CA, USA) and then analyzed using a flow cytometer (FACSVerse, BD Biosciences). The percentages of positive cells were determined by comparison with the corresponding control cells stained with an isotype-matched antibody, in which a false-positive rate of less than 1 % was acceptable. The isolated cells were positive for CD73, CD90, CD105, and CD146, and negative for CD11b, CD14, CD35, and CD45 (data not shown). Furthermore, P3 cells were cultured under osteogenic, chondrogenic, or adipogenic conditions [17, 18], and showed differentiation capacities for osteoblasts (odontoblasts in the case of SHED), chondrocytes, and adipocytes (data not shown). These findings showed that our isolated cells were MSCs based on the standard MSC criteria [22].

### Systemic MSC transplantation into MRL/*lpr* mice

P3 hBMMSCs and SHED were collected and washed with PBS three times. The donor cells were diluted in PBS, and intravenously infused at 1×10^5^ per 10 g body weight into 16-week-old MRL/*lpr* mice via the right cervical vein according to a previously published method [16]. The mice were analyzed at 20 weeks of age. Age-matched MRL/*lpr* mice that received PBS were used as controls.

### Enzyme-linked immunosorbent assays of biological (blood serum and bone marrow) and culture (culture supernatant) samples

C-terminal telopeptides of type I collagen (CTX), interleukin (IL)-17, and soluble receptor activator for nuclear factor κB (NF-κB) ligand (sRANKL) in the samples were measured using enzyme-linked immunosorbent assay (ELISA) kits (IL-17 and sRANKL, R&D Systems, Minneapolis, MN, USA; CTX, Nordic Bioscience Diagnostics A/S, Herlev, Denmark) according to the manufacturer’s instructions.

### Microcomputed tomographic bone analysis

Femoral bones of mice were analyzed by microcomputed tomography (microCT) with a 1076 microCT system (Skyscan, Kontich, Belgium), as previously described [23]. Density values were calibrated using hydroxyl apatite phantoms with bone mineral density (BMD) values of 0.25 and 0.75 g/cm^3^ (Skyscan). BMD and bone structural indices (bone volume/trabecular volume (BV/TV), trabecular thickness (Tb.Th), trabecular number (Tb.N), and trabecular separation (Tb.Sp)) were calculated.

### Histological bone analysis

Tibias were fixed with 4 % paraformaldehyde in PBS and decalcified with 10 % ethylenediaminetetraacetic acid. Paraffin sections were prepared at a thickness of 6 μm and stained with tartrate-resistant acid phosphate (TRAP) [21]. The number of TRAP-positive cells per total bone area in the bone metaphysis was analyzed in five representative images by Image J software (National Institutes of Health, Bethesda, MA, USA).

### Culture and stimulation of mouse BMCs

Mouse BMCs were cultured in Dulbecco’s modified Eagle’s medium (Nacalai Tesque) supplemented with 10 % FBS, 50 μM 2-mercaptoethanol (Invitrogen), 10 mM HEPES (Nacalai Tesque), 1 mM sodium pyruvate (Nacalai Tesque), 1 % non-essential amino acid (Nacalai Tesque), 2 mM L-glutamine, 100 U/ml penicillin, and 100 μg/ml streptomycin with plate-bound anti-CD3 (1 μg/ml; eBioscience) and soluble anti-CD28 (1 μg/ml; eBioscience) antibodies on 24-well plates. The conditioned medium (CM) was collected and enriched ten-fold.

### Th17 cell assay of cultured mouse BMCs

Cultured BMCs were flashed out from mouse long bones (femurs and tibias), and treated with 0.82 % NH_4_Cl in PBS for 15 minutes at room temperature. The BMCs (1×10^5^ in 100 μl) were then incubated with PerCP-conjugated anti-CD4 antibody (1 μg; eBioscience) and then R-PE-conjugated anti-IL-17 (eBioscience) and APC-conjugated anti-interferon (IFN) gamma (eBioscience) antibodies (1 μg each). Isotype-matched antibodies (eBioscience) were used as controls. The number of CD4^+^IL-17^+^IFNγ^−^ Th17 cells per 1×10^4^ cells was measured on the flow cytometer.

### Isolation and culture of mouse BMMSCs

Mouse BMMSCs were isolated based on the CFU-F method [23]. BMCs were seeded at 1–2×10^7^ cells per 100-mm culture dish. After 3 h, the cells were washed with PBS twice to eliminate non-adherent cells, and the attached cells were cultured for 14–16 days. Attached colonies consisting of spindle-shaped cells were observed under a microscope. The colony-forming attached cells were passaged once. The cells were cultured in αMEM supplemented with 20 % FBS, 2 mM L-glutamine, 55 μM 2-mercaptoethanol, 100 U/ml penicillin, and 100 μg/ml streptomycin. Based on the MSC criteria [22], the colony-forming cells were characterized as described previously [24]: 1) flow cytometry demonstrated immunophenotypes of CD73, CD105, CD146, Sca-1, and SSEA-4, and negativity for CD14, CD34, and CD45; 2) mouse BMMSCs were evaluated for differentiation into osteoblasts, chondrocytes, and adipocytes under the corresponding specific culture conditions.

### In vitro osteogenic capacity of mouse BMMSCs

Mouse BMMSCs were cultured under osteogenic conditions. The osteogenic medium consisted of αMEM containing 20 % FBS, 2 mM L-glutamine, 55 μM 2-mercaptoethanol, 100 μM L-ascorbic acid 2-phosphate (Wako Pure Chemical Industrials), 2 mM β-glycerophosphate (Sigma, St. Louis, MO, USA), 10 nM dexamethasone (Sigma), 100 U/ml penicillin, and 100 μg/ml streptomycin. The cultures performed in the presence or absence of CM of mouse BMCs and 10 nM recombinant mouse IL-17 (R&D Systems) with or without 1 μg/ml rat anti-mouse IL-17 IgG_2A_ antibody (R&D Systems) or the control antibody, rat IgG_2A_ antibody (R&D Systems), for 4 weeks. The ten-fold-enriched CM was mixed with the osteogenic medium at a ratio of 1:9. The cultures were stained with 1 % Alizarin Red solution [21]. Alizarin Red-positive areas were quantified by Image J software [21].

### In vitro osteoclast assay

We co-cultured mouse BMCs (1×10^6^/well) with mouse calvarial cells (1×10^5^/well) or mouse BMMSCs (1×10^5^/well). Some mouse BMMSCs were pretreated with CM of mouse BMCs or 10 nM recombinant mouse IL-17 in the presence 1 μg/ml anti-mouse IL-17 antibody or the control antibody for 3 days. Some mouse BMCs (1×10^6^/well) were pretreated with IL-17 and control siRNAs (53 nM; Santa Cruz Biotechnology, Dallas, TX, USA) according to the manufacturer’s instruction for 24 h at 37 °C and then continued to be cultured in the above medium. The ten-fold-enriched CM was mixed with the growth medium at a ratio of 1:9. Mouse calvarial bones were harvested from 2–3-day-old wild-type C57BL/6 mice and the calvarial cells were isolated with a sequential enzymatic method [23]. The osteoclastogenic medium consisted of αMEM containing 10 % FBS, 100 U/ml penicillin, 100 μg/ml streptomycin, 10 nM vitamin D_3_ (Wako Pure Chemical Industrials), and 1 nM prostaglandin E_2_ (Wako Pure Chemical Industrials) for 7 days. BMCs were also stimulated with 10 ng/ml macrophage-colony stimulating factor (M-CSF; R&D Systems) and 50 ng/ml RANKL (PeproTec, Rocky Hill, NJ, USA) for 5 days. The cultures were treated with TRAP staining [23]. TRAP-positive multinucleated cells (MNCs) (>3 nuclei) were determined as osteoclast-like cells [23].

### Western blotting

All the samples were lyzed in M-PER mammalian protein extraction reagent (Thermo, Rockford, IL, USA) containing proteinase inhibitor cocktail (Nacalai Tesque). They were separated by sodium dodecyl sulfate-polyacrylamide gel and transferred to Immobilon-P membranes (Millipore, Billerica, MA, USA). The membranes were blocked with 5 % skimmed milk in Tris-buffered saline (150 mM NaCl and 20 mM Tris–HCl, pH 7.2) for 1 h at room temperature and then incubated with anti-mouse IL-17 antibody overnight at 4 °C. They were then treated with horseradish peroxidase-conjugated donkey anti-rabbit or anti-mouse IgG antibody (1:1000; Santa Cruz Biotechnology) for 1 h at room temperature. The bound antibodies were visualized using SuperSignal West Pico (Thermo).

### Statistical analysis

Data were analyzed by the one-way analysis of variance F-test. *P*-values of less than 0.05 were considered to be significant.

### Supplementary materials and methods

Supplementary materials and methods are provided as Additional file 1.

## Results

### Systemic human MSC transplantation ameliorates SLE disorders in MRL/*lpr* mice

According to a previous method [16], hBMMSCs and SHED (1×10^5^ per 10 g body weight) were systemically transplanted through the right cervical vein into MRL/*lpr* mice at 16 weeks of age (Fig. S1a in Additional file 2), following which they exhibited severe autoimmune disorders including abnormal autoantibody increments and severe renal nephritis (Fig. S1b and c in Additional file 2) [10]. The systemic transplantation of human MSCs was followed by evaluation of their immune therapeutic efficacy in the SLE-like disorders, such as hyper-autoimmune antibody production and renal dysfunction in MRL/*lpr* mice at 4 weeks post-transplantation (Fig. S1b and c in Additional file 2) as described previously [16, 17].

### Systemic human MSC transplantation improves secondary bone loss in MRL/*lpr* mice

**Fig. 1:**
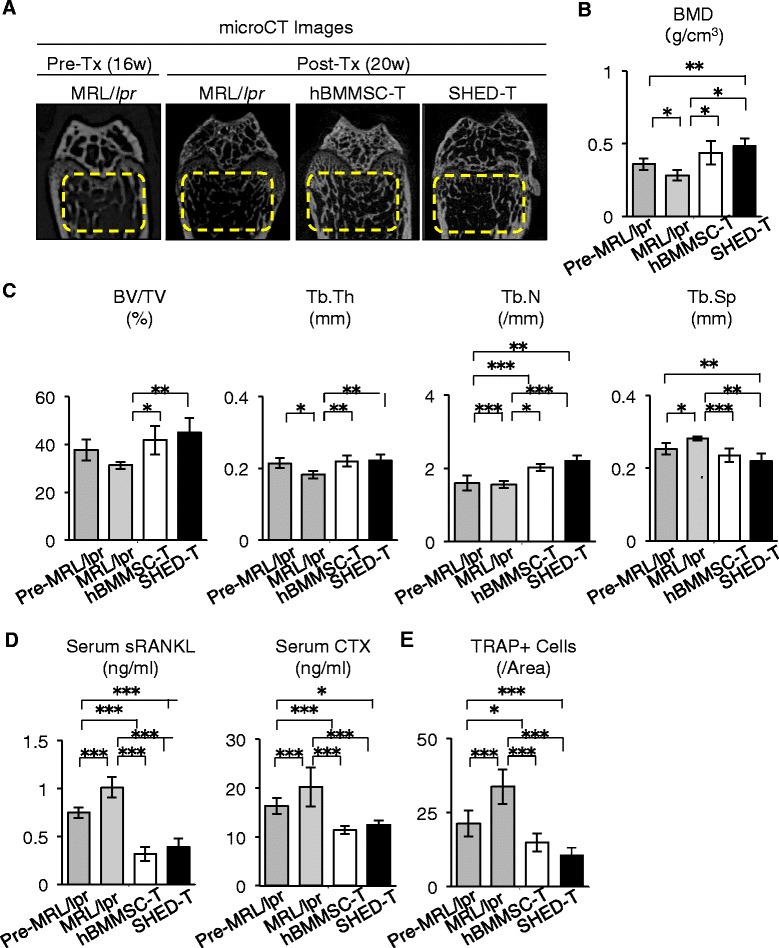
Systemic human MSC transplantation ameliorates the bone loss in MRL/*lpr* mice. **a**–**c** Morphological analyses of mouse tibias. **a** Representative micro-computed tomography (*microCT*) images of trabecular bone structures (yellow-dashed areas). *Pre-Tx (16w)* pre-transplant stage of MRL/*lpr* mice at 16 weeks of age; *Post-Tx (20w)* post-transplant stage of MRL/*lpr* mice at 20 weeks of age. **b** Bone mineral density (BMD). **c** Trabecular bone parameter assay. *BV/TV* bone volume ratio to tissue volume; *Tb.N* trabecular number; *Tb.Sp* trabecular separation; *Tb.Th* trabecular thickness. **d**, **e** In vivo osteoclast activity assay. **d** ELISA analyses of mouse serum. *CTX* C-terminal telopeptides of type I collagen; *sRANKL* soluble RANKL. **e** Histological analysis of recipient tibias by TRAP staining. *TRAP+ cells* TRAP-positive osteoclast-like cells. *hBMMSC-T* human bone marrow mesenchymal stem cell-transplanted MRL/*lpr* mice at 20 weeks; *Pre-MRL/lpr* pre-transplant MRL/*lpr* mice at 16 weeks of age; *SHED-T* stem cells from exfoliated deciduous teeth-transplanted MRL/*lpr* mice at 20 weeks. n = 5 for all groups. *P < 0.05, **P < 0.01, ***P < 0.005. Values are shown as means ± SD

Severe osteoporosis with progressive trabecular bone breakdown occurs secondarily in SLE model MRL/*lpr* mice at 16 weeks of age (Fig. 1) [10] and SLE patients [4]. To investigate effects of transplanted human MSC on skeletal disruption in the SLE model mice, we first examined the detailed in vivo skeletal metabolism in MRL/*lpr* mice at 4 weeks after hBMMSCs and SHED transplantation. MicroCT and histological analyses showed that systemic transplantation of hBMMSCs and SHED recovered the BMD and trabecular bone structures in MRL/*lpr* mice (Fig. 1a–c, Fig. S2a in Additional file 2). Real-time reverse transcription polymerase chain reaction (RT-PCR) demonstrated that the long bones of human MSC-transplanted MRL/*lpr* mice expressed higher levels of osteoblast-specific genes (*runt-related transcription factor 2* (*Runx2*), alkaline phosphatase (*Alp*), and *osteocalcin*) than those in non-transplanted MRL/*lpr* mice (Fig. S2b in Additional file 2). Furthermore, compared with non-transplanted mice, human MSC transplantation markedly reduced the serum bone resorption markers sRANKL and CTX as shown by ELISA (Fig. 1d) and the number of TRAP-positive cells in the epiphysis of long bones as demonstrated by histological analysis (Fig. 1e, Fig. S2c in Additional file 2). These findings suggested that human MSC transplantation recovered the bone reduction in MRL/*lpr* mice via accelerated bone formation and suppressed bone resorption, but did not elucidate the detailed therapeutic mechanisms at cellular and molecular levels.

### Systemic human MSC transplantation recovers dysregulation of osteoblast and osteoclast development via recipient BMMSCs in MRL/*lpr* mice

**Fig. 2:**
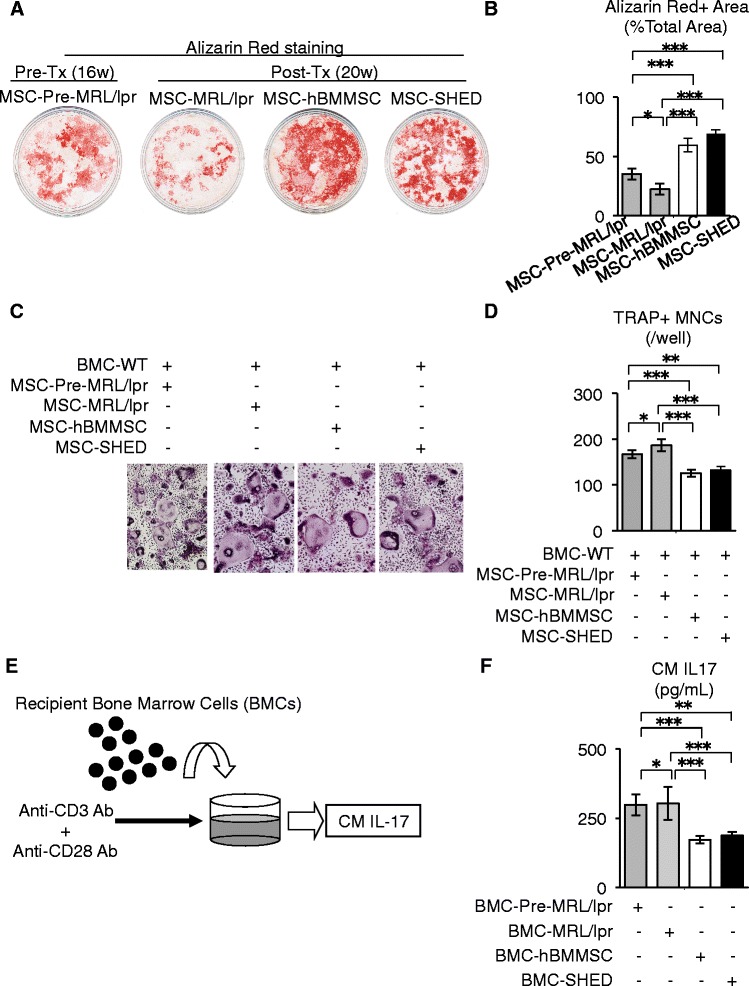
Systemic human MSC transplantation recovers bone metabolism and IL-17 production in bone marrow of MRL/*lpr* mice. **a**, **b** Osteogenic capacity of recipient BMMSCs. Alizarin Red staining at 4 weeks after osteogenic induction. **c**, **d** Osteoclast inductivity of recipient BMMSCs in co-culture with wild-type BL/6 mice-derived bone marrow cells (*BMC-WT*) under stimulation with 1α, 25-(OH)_2_ vitamin D_3_ and prostaglandin E_2_. TRAP staining. *TRAP+ MNCs* tartrate-resistant acid phosphate-positive multinucleated cells. **e**, **f** Production of interleukin-17 (IL-17) in recipient bone marrow cells (*BMCs*). **e** A schema of recipient BMC culture. BMCs were cultured under stimulation with anti-CD3 (*Anti-CD3 Ab*) and anti-CD28 (*Anti-CD28 Ab*) antibodies, and then IL-17 was measured in the conditioned medium (*CM IL-17*). **f** ELISA of CM IL-17. *BMC-hBMMSC* bone marrow cells isolated from human bone marrow mesenchymal stem cell-transplanted MRL/*lpr* mice at 20 weeks of age; *BMC-MRL/lpr* bone marrow cells isolated from non-transplanted MRL/*lpr* mice at 20 weeks of age; *BMC-Pre-MRL/lpr*, bone marrow cells isolated from pre-transplant MRL/*lpr* mice at 16 weeks of age; *BMC-SHED* bone marrow cells isolated from stem cells from exfoliated deciduous teeth-transplanted MRL/*lpr* mice at 20 weeks of age; *MSC-hBMMSC* bone marrow mesenchymal stem cells isolated from human bone marrow mesenchymal stem cell-transplanted MRL/*lpr* mice at 20 weeks of age; *MSC-MRL/lpr* bone marrow mesenchymal stem cells isolated from non-transplanted MRL/*lpr* mice at 20 weeks of age; *MSC-Pre-MRL/lpr* bone marrow mesenchymal stem cells isolated from pre-transplant MRL/*lpr* mice at 16 weeks of age; *MSC-SHED* bone marrow mesenchymal stem cells isolated from stem cells from exfoliated deciduous teeth-transplanted MRL/*lpr* mice at 20 weeks of age; *Pre-Tx (16w)* pre-transplant stage of MRL/*lpr* mice at 16 weeks of age; *Post-Tx (20w)* post-transplant stage of MRL/*lpr* mice at 20 weeks of age. n = 5 for all groups. *P < 0.05, **P < 0.01, ***P < 0.005. Values are shown as means ± SD

Dysregulation of osteoblast and osteoclast development in bone marrow leads to skeletal dysfunction. BMMSCs play a crucial role in the development of osteoblasts and osteoclasts in bone marrow [25–27]. Recent studies showed that recipient BMMSCs in SLE mice and patients impaired the stemness and osteogenic capacity [10, 19]. Therefore, we hypothesized that recipient BMMSCs might participate in the osteoporosis of SLE and may be a therapeutic target. We isolated recipient BMMSCs from non-, hBMMSC-, and SHED-transplanted MRL/*lpr* mice, and designated them as MSC-MRL/*lpr*, MSC-hBMMSC, and MSC-SHED, respectively. We also isolated recipient BMMSCs from pre-transplant MRL/*lpr* mice at 16 weeks of age (designated as MSC-Pre-MRL/*lpr*). MSC-hBMMSC and MSC-SHED showed lower CFU-F numbers, a higher population-doubling score, and an elevated cell proliferation rate compared with those of MSC-MRL/*lpr*, as well as MSC-Pre-MRL/*lpr* (Fig. S3 in Additional file 2). Next, we examined the osteogenic capacity of recipient BMMSCs. Alizarin Red staining showed markedly lower mineral deposition by MSC-Pre-MRL/*lpr* and MSC-MRL/*lpr* than that by wild-type mouse-derived MSCs (MSC-WT) at 4 weeks after osteogenic induction (data not shown). Compared with MSC-Pre-MRL/*lpr* and MSC-MRL/*lpr*, MSC-hBMMSC and MSC-SHED showed enhanced mineral accumulation (Fig. 2a and b) and osteoblast-specific gene expression (Fig. S4a in Additional file 2).

Furthermore, we examined the effects of human MSC transplantation on recipient BMMSC-dependent osteoclastic differentiation. Recipient BMMSCs were co-cultured with BMCs derived from wild-type mice (BMC-WT) under stimulation with vitamin D_3_ and prostaglandin E_2_. TRAP staining demonstrated that MSC-MRL/*lpr*, as well as MSC-Pre-MRL/*lpr*, showed enhanced TRAP-positive MNCs compared with that of MSC-WT (data not shown). Moreover, compared with MSC-Pre-MRL/*lpr* and MSC-MRL/*lpr*, this co-culture assay showed that MSC-hBMMSC and MSC-SHED had remarkably reduced capacities for differentiation into TRAP-positive MNCs (Fig. 2c) and suppressed expression of osteoclast-specific genes (*nuclear factor of activated T-cells (Nfatc1), calcitonin receptor*, and *cathepsin K*) (Fig. S4b in Additional file 2). Taken together, these data suggested that hBMMSC and SHED transplantation improved the dysfunction of recipient BMMSCs by induction of osteoblasts and suppression of osteoclasts to regenerate abnormal skeletal structures in MRL/*lpr* mice.

### Systemic human MSC transplantation suppresses abnormal IL-17 production in the bone marrow of recipient MRL/*lpr* mice

The proinflammatory cytokine IL-17 is produced and secreted by a subset of helper T cells called Th17 cells, and contributes to the autoimmune progression in SLE [28, 29]. MRL/*lpr* mice express a significant increment of systemic Th17 cells and IL-17 levels when compared to wild-type C57/BL6 mice (data not shown), as shown previously [10]. We examined the immunoregulatory effect of hBMMSCs and SHED on IL-17 production using a co-culture system of human MSCs with human T cells in the presence of transforming growth factor-β_1_ and IL-6. This co-culture system demonstrated that hBMMSCs and SHED suppressed both Th17 cell differentiation and IL-17 secretion by flow cytometric analysis and ELISA, respectively (Fig. S5a and b in Additional file 2). To confirm in vivo immunological effects of hBMMSCs and SHED on IL-17 secretion, we analyzed peripheral levels of Th17 cells and IL-17 in MRL/*lpr* mice. Immunological analyses showed that hBMMSCs and SHED suppressed systemic Th17 cells and IL-17 expression in MRL/*lpr* mice, as well as pre-transplant MRL/*lpr* mice (Fig. S5c and d in Additional file 2). These findings indicated that hBMMSCs and SHED expressed a suppressive regulation of Th17 cell differentiation in vitro and in vivo, as reported previously [10, 16].

Carboxyfluorescein diacetate succinimidyl ester (CFSE)-labeled hBMMSCs and SHED were infused intravenously into MRL/*lpr* mice. Subsequently, these cells were found in the recipient bone marrow space at days 1 and 7 after infusion, but the positive cell number was lower at day 7 than that at day 1 (Fig. S6a in Additional file 2). IL-17 is significantly increased in the bone marrow of MRL/*lpr* mice [10]. We analyzed IL-17 levels in bone marrow tissues of hBMMSC- and SHED-transplanted MRL/*lpr* mice in comparison with non-transplanted MRL/*lpr* mice. In immunofluorescence analysis, hBMMSC and SHED transplantation resulted in a marked decrease in IL-17-positive cells in the recipient bone marrow (Fig. S6b in Additional file 2). Flow cytometric analysis and ELISA showed reductions of CD4^+^IL-17^+^IFNγ^−^ Th17 cells and IL-17 in the recipient bone marrow tissues of hBMMSC- and SHED-transplanted groups, respectively, when compared to pre-transplant and non-transplanted MRL/*lpr* mice (Fig. S6c and d in Additional file 2). To assess the productivity of IL-17 in recipient bone marrow, we individually cultured the four types of recipient BMCs (BMC-Pre-MRL/*lpr*, BMC-MRL/*lpr*, BMC-hBMMSC, and BMC-SHED) from pre-transplant, and non-, hBMMSC- and SHED-transplanted MRL/*lpr* mice, respectively, under T cell activation conditions stimulated with anti-CD3 and anti-CD28 antibodies for 3 days and then measured IL-17 in the CMs by ELISA (Fig. 2e). BMC-hBMMSC and BMC-SHED exhibited lower production of IL-17 than BMC-MRL/*lpr*, as well as BMC-Pre-MRL/*lpr* (Fig. 2f), reflecting the recipient bone marrow IL-17 conditions. Taken together, these findings indicated that transplanted hBMMSCs and SHED might suppress the abnormal IL-17 production in the recipient bone marrow of MRL/*lpr* mice, but did not evaluate whether the recipient IL-17 conditions effected on the dysregulation by osteoclastic bone resorption and osteoblastic bone formation in MRL/*lpr* mice.

To understand the mechanistic transplant studies, we transplanted hBMMSCs and SHED (1×10^5^ per 10 g body weight) into wild-type C57BL/6 mice at 16 weeks of age, and analyzed the effects of the transplants 4 weeks after the infusion (Fig. S1a in Additional file 2). There was no effect of hMSC transplantation on bone metabolism and IL-17 levels (Fig. S7 in Additional file 2). These findings suggested that recipient inflammatory conditions might influence the transplants’ ability to treat recipient bone metabolism.

### Systemic human MSC transplantation rescues impaired osteogenic capacity of recipient BMMSCs through IL-17-dependent immune conditions in the recipient bone marrow of MRL/*lpr* mice

**Fig. 3:**
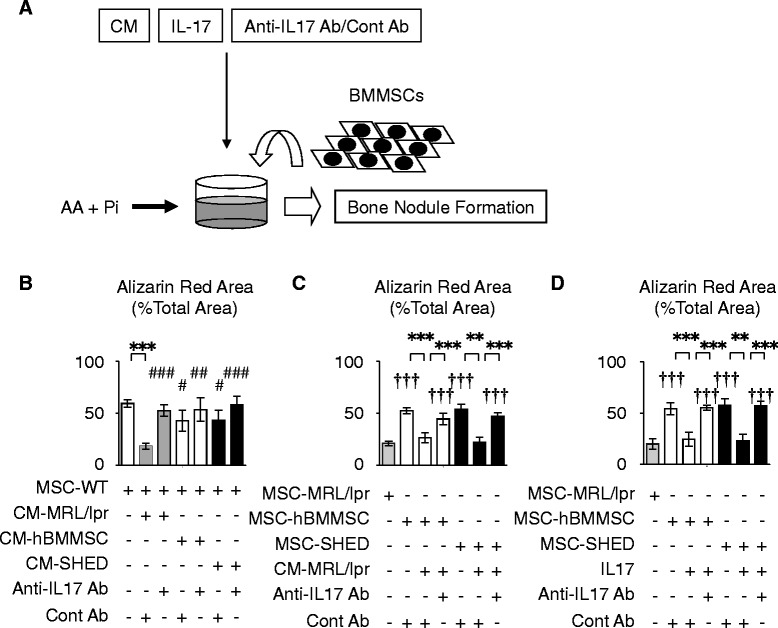
Systemic human MSC transplantation recovers IL-17-impaired recipient BMMSC-mediated osteogenesis in the bone marrow of MRL/*lpr* mice. **a** A schema of the osteogenic capacity of recipient bone marrow mesenchymal stem cells (*BMMSCs*). Recipient BMMSCs were induced by ascorbic acid (*AA*) and inorganic phosphate (*Pi*), and treated with or without recipient BMC-derived conditioned medium (*CM*) in the presence of anti-mouse IL-17 antibody (*Anti-IL-17 Ab*), control antibody for anti-mouse IL-17 antibody (*Cont Ab*) or recombinant mouse IL-17 (*IL-17*). **b**–**d** Osteogenic assay by Alizarin Red staining at 4 weeks after osteogenic induction. n=5 for all groups. Values are shown as means ± SD. **P < 0.01, ***P < 0.005. ^#^ *P* < 0.05, ^##^ *P* < 0.01, ^###^ *P* < 0.005, versus MSC-WT treated with CM-MRL/lpr in the presence of Cont Ab. ^†††^ *P* < 0.005, versus MSC-MRL/lpr. *CM-hBMMSC* conditioned medium of BMC-hBMMSC culture under stimulation with anti-CD3 and CD28 antibodies; *CM-MRL/lpr*, conditioned medium of BMC-MRL/lpr culture under stimulation with anti-CD3 and CD28 antibodies; *CM-SHED* conditioned medium of BMC-SHED culture under stimulation with anti-CD3 and CD28 antibodies; *MSC-WT* BMMSCs isolated from wild-type C57BL/6 mice

In preliminary examinations, MSC-WT were cultured under osteogenic conditions in the presence of IL-17 and CM of BMC-MRL/*lpr* (CM-MRL/*lpr*) (Fig. S8a and b in Additional file 2). The treatments of IL-17 and CM-MRL/*lpr* led to a significant reduction in the osteogenic capacity of MSC-WT as demonstrated by Alizarin Red staining (Fig. S8a and b in Additional file 2). Anti-IL-17 antibody treatment reversed the suppressed osteogenic capacity in IL-17- and CM-MRL/*lpr*-treated MSC-WT when co-treated with the control antibody treatment (Fig. S8a and b in Additional file 2). These preliminary data suggested that IL-17 conditions in the recipient bone marrow might modulate the osteoblastic differentiation of recipient BMMSCs in MRL/*lpr* mice. We then examined whether IL-17-dependent immune conditions in recipient bone marrow affected recipient BMMSC-mediated bone formation. Mouse BMMSCs were cultured under osteogenic conditions with or without CM collected from BMC-hBMMSC and BMC-SHED cultures (CM-hBMMSC, and CM-SHED, respectively), as well as CM-MRL/*lpr*, in the presence or absence of anti-mouse IL-17 antibody (Fig. 3a). The control antibody for anti-mouse IL-17 antibody was used as the control treatment. Alizarin Red staining results showed that CM-MRL/*lpr* significantly suppressed the osteogenic capacity of MSC-WT (Fig. 3b, Fig. S8b in Additional file 2), whereas CM-hBMMSC and CM-SHED showed little suppressive effects on the osteogenic capability of MSC-WT (Fig. 3b). Anti-IL-17 antibody treatment neutralized the inhibited osteogenic capability by the individual CMs, especially in the CM-MRL/*lpr*-treated group (Fig. 3b, Fig. S8b in Additional file 2). These results suggested that the hyperactivity of IL-17 in recipient bone marrow of MRL/*lpr* mice might cause the severe defective bone formation mediated by recipient BMMSCs.

Next, we investigated the effect of abnormal IL-17 in recipient bone marrow of MRL*/lpr* mice on recipient BMMSC-mediated bone formation. Recipient BMMSCs, including MSC-MRL/*lpr*, MSC-hBMMSC and MSC-SHED, were treated with CM-MRL/*lpr* under osteogenic conditions. CM-MRL/*lpr* treatment significantly suppressed the mineralized deposition induced by MSC-hBMMSC and MSC-SHED (Fig. 3c). The suppressed MSC-hBMMSC- and MSC-SHED-induced mineralization showed similar rates to MSC- MRL/*lpr*-induced bone formation (Fig. 3c). The CM-MRL/*lpr* treated suppression was recovered by the treatment with anti-IL-17 antibody (Fig. 3c). We then cultured individual recipient BMMSCs under osteogenic conditions in the presence of IL-17. IL-17 significantly suppressed the mineralized deposition induced by MSC-hBMMSC and MSC-SHED, which showed similar deposition rates to that of MSC-MRL/*lpr* (Fig. 3d). Anti-IL-17 antibody treatment completely neutralized the IL-17-suppressed osteogenic capacities of MSC-hBMMSC and MSC-SHED (Fig. 3d). These findings indicated that abnormal IL-17 in the bone marrow of MRL/*lpr* mice impaired the osteogenic capacity of recipient BMMSCs, and suggested that hBMMSC and SHED transplantation recovered the osteogenic dysfunction of recipient BMMSCs through inhibiting hyperactivated IL-17 in the recipient bone marrow of MRL/*lpr*.

### Systemic human MSC transplantation rescues impaired recipient BMMSC-mediated osteoclast induction through IL-17-dependent immune conditions in the recipient bone marrow of MRL/*lpr* mice

**Fig. 4:**
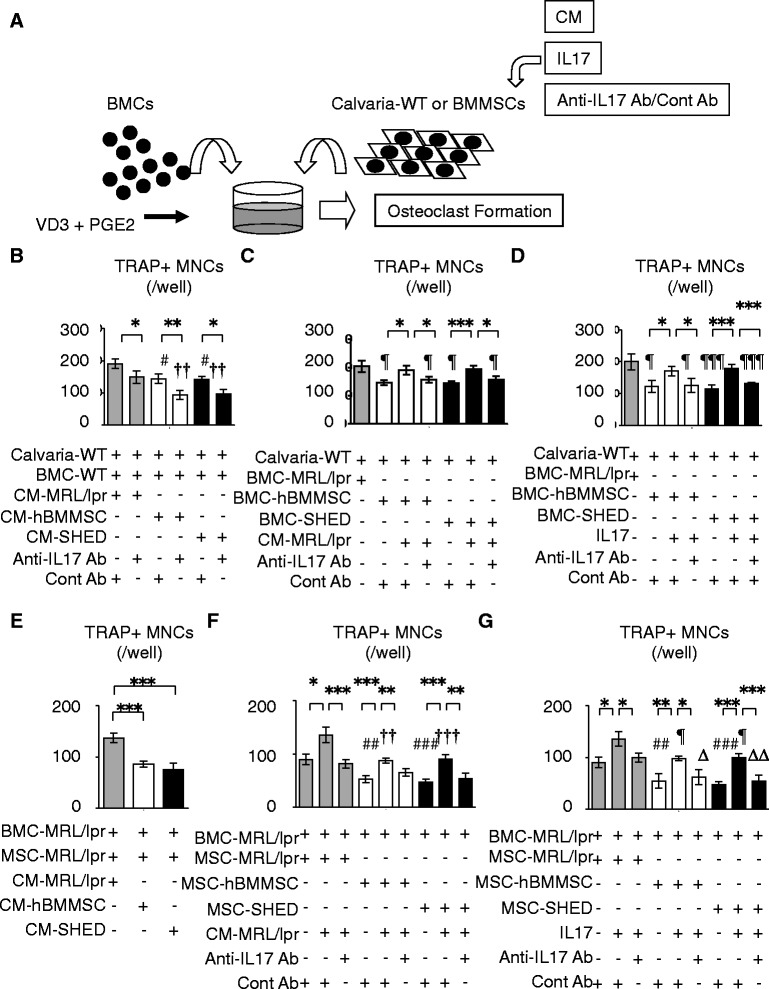
Systemic human MSC transplantation recovers IL-17-accelerated recipient BMMSC-mediated osteoclastogenesis in the bone marrow of MRL/*lpr* mice. **a** A schema of osteoclastic induction. Mouse bone marrow cells (*BMCs*) were co-cultured with wild-type mouse-derived calvarial cells (*Calvaria-WT*) or recipient bone marrow mesenchymal stem cells (*BMMSCs*) under stimulation with 1α, 25-(OH)_2_ vitamin D_3_ (*VD3*) and prostaglandin E_2_ (*PGE2*). Calvaria-WT and recipient BMMSCs were pretreated with or without recipient BMC-derived conditioned medium (*CM*) or interleukin-17 (*IL17*) in the presence of anti-mouse IL-17 antibody (*Anti-IL-17 Ab*) or control antibody for anti-mouse IL-17 antibody (*Cont Ab*). **b**–**g** Osteoclast induction assay by tartrate-resistant acid phosphate (*TRAP*) staining. n = 5 for all groups. Values are shown as means ± SD. *P < 0.05, **P < 0.01, ***P < 0.005. **b** Co-culture of Calvaria-WT and wild-type mice-derived bone marrow cells (*BMC-WT*). ^#^ *P* < 0.05, versus CM-MRL/*lpr* pretreatment in the presence of Cont Ab; ^††^ *P* < 0.01, versus CM-MRL/*lpr* pretreatment in the presence of Anti-IL-17 Ab. **c**, **d** Co-culture of Calvaria-WT and recipient BMCs. ^¶^ *P* < 0.05, ^¶¶^ *P* < 0.01, ^¶¶¶^ *P* < 0.005, versus BMC-MRL/*lpr* and Calvaria-WT co-culture. **e** Co-culture of BMC-MRL/lpr and MSC-MRL/lpr. **f**, **g** Co-culture of BMC-MRL/lpr and recipient BMMSCs. ^##^ *P* < 0.01, ^###^ *P* < 0.005, versus co-culture of BMC-MRL/lpr and Cont Ab-pretreated MSC-MRL/lpr . **f** ^††^ *P* < 0.01, ^†††^ *P* < 0.005 versus co-culture of BMC-MRL/lpr and CM-MRL/lpr-pretreated MSC-MRL/lpr. **g** ^¶^ *P*<0.05, versus co-culture of BMC-MRL/*lpr* and IL-17- and Cont Ab-pretreated MSC-MRL/*lpr* co-culture pretreated with IL-17; ^∆^ *P* < 0.05, ^∆∆^ *P* < 0.01, versus co-culture of BMC-MRL/*lpr* and IL-17- and Anti-IL-17 Ab-pretreated MSC-MRL/*lpr. MNC* multinucleated cell; *MSC* mesenchymal stem cell; *SHED* stem cells from exfoliated deciduous teeth

We also preliminarily examined the effects of IL-17 and CM-MRL/*lpr* on osteoclast differentiation in the co-culture system with BMC-WT and wild-type mouse-derived calvarial cells (Calvaria-WT). BMC-WT were co-cultured with Calvaria-WT under the stimulation of vitamin D_3_ and prostaglandin E_2_ in the presence or absence of IL-17 and CM-MRL/*lpr* (Fig. 4a). The control antibody for anti-mouse IL-17 antibody was also used as the control treatment. TRAP staining showed that both IL-17 and CM-MRL/*lpr* treatments induced TRAP-positive MNCs in the co-culture system (Fig. S8c and d in Additional file 2). The enhanced TRAP-positive MNC-induction was reversed when treated with anti-IL-17 antibody in the presence of IL-17 and CM-MRL/*lpr* (Fig. S8c and d in Additional file 2). In the co-culture system of BMC-WT and Calvaria-WT, CM-hBMMSC and CM-SHED treatments showed a fewer number of TRAP-positive MNCs than CM-MRL/*lpr* treatment (Fig. 4b). Anti-IL-17 antibody treatment reduced the number of TRAP-positive MNCs under the stimulation of CM-hBMMSC and CM-SHED (Fig. 4b). Subsequently, we co-cultured Calvaria-WT with recipient BMCs. Induction of TRAP-positive MNCs in the co-culture systems with BMC-hBMMSC and BMC-SHED showed a fewer number of TRAP-positive MNCs than that with BMC-MRL/*lpr* (Fig. 4c and d). Treatment with CM-MRL/*lpr* and IL-17 stimulated the reduced osteoclast induction in the co-culture systems with BMC-hBMMSC and BMC-SHED (Fig. 4c and d). Anti-IL-17 antibody treatment neutralized the CM-MRL/*lpr*- and IL-17-enhanced TRAP-positive MNC-induction in the co-culture systems with BMC-hBMMSC and BMC-SHED (Fig. 4c and d). These results suggested that IL-17-dependent immune conditions in the recipient bone marrow might modulate osteoclastic differentiation and bone resorption in MRL/*lpr* mice.

To examine whether IL-17-dependent immune conditions in recipient bone marrow affect recipient BMMSC-mediated osteoclast induction, we pretreated recipient BMMSCs with CM of recipient BMC culture or IL-17 in the presence or absence of anti-IL-17 antibody. In the co-culture system of MSC-MRL/*lpr* and BMC-MRL/*lpr*, TRAP staining demonstrated that CM-MRL/*lpr*-enhanced TRAP-positive MNC induction was reduced when pretreated with CM-hBMMSC and CM-SHED (Fig. 4e). BMC-MRL/*lpr* were then co-cultured with individual recipient MSCs. MSC-hBMMSC and MSC-SHED showed less TRAP-positive MNC-inductive functions in comparison with MSC-MRL/*lpr* (Fig. 4f and g). CM-MRL/*lpr*-pretreated recipient BMMSCs significantly enhanced the TRAP-positive MNC induction from BMC-MRL*/lpr* in the respective co-culture system, but the pretreatment effects on MSC-hBMMSC and MSC-SHED were less than those on MSC-MRL/*lpr* (Fig. 4f). Anti-IL-17 antibody co-pretreatment blocked the CM-MRL/*lpr*-enhanced TRAP-positive MNCs in the individual co-culture system (Fig. 4f). We then examined whether IL-17 treatment in recipient BMMSCs directly affected the osteoclast inductivity in the co-culture system with BMC-MRL/*lpr*. IL-17-pretreated MSC-hBMMSC and MSC-SHED, as well as MSC-MRL/*lpr*, significantly induced TRAP-positive MNCs, but the inductivity by IL-17-pretreated MSC-hBMMSC and MSC-SHED was less than that by IL-17-pretreated MSC-MRL/*lpr* (Fig. 4g). The increased TRAP-positive MNC inductivity was inhibited when the recipient BMMSCs were co-pretreated with anti-IL-17 antibody (Fig. 4g). These findings suggested that IL-17-dependent hyperimmune conditions in the recipient bone marrow of MRL/*lpr* mice might impair recipient BMMSCs to accelerate abnormal osteoclast induction while human MSC transplantation might target the impaired recipient BMMSCs to suppress the enhanced osteoclast inductivity.

### Functional downregulation of IL-17 in BMCs of MRL/*lpr* mice inhibits recipient BMMSC-mediated osteoclast differentiation

**Fig. 5:**
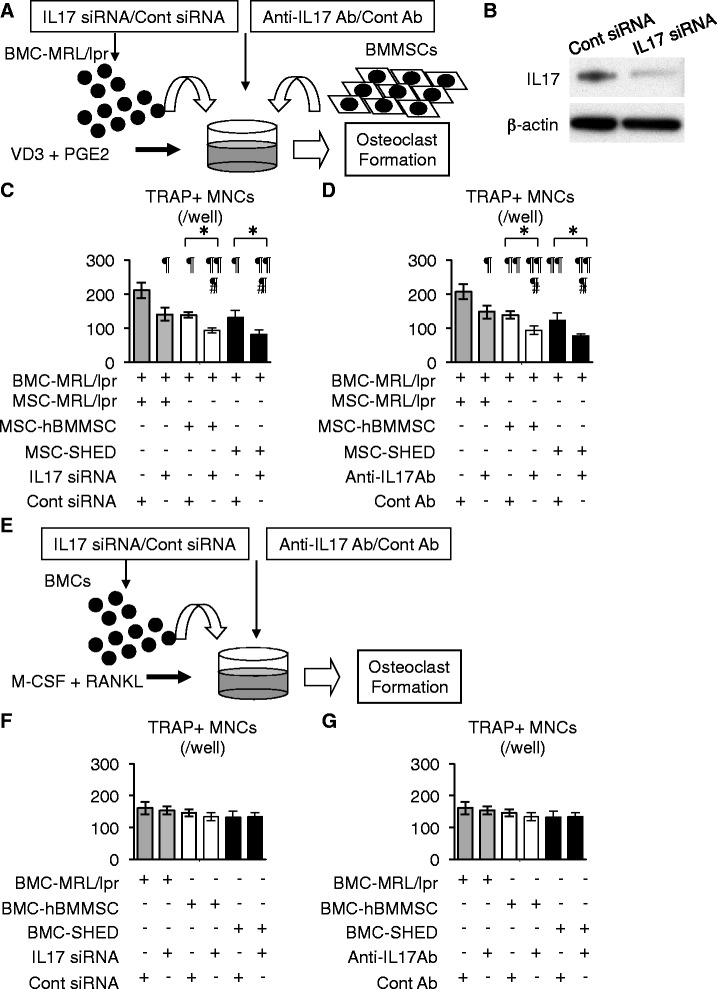
Functional downregulation of IL-17 in bone marrow cells of MRL/*lpr* mice suppressed recipient BMMSC-mediated osteoclastogenesis. **a**–**d** Osteoclast induction assay in bone marrow cell MRL/lpr (*BMC-MRL/lpr*) and recipient bone marrow mesenchymal stem cell (*BMMSC*) co-culture. **a** A schema of osteoclast induction in a co-culture system. BMC-MRL/lpr were pretreated with or without mouse interleukin-17 siRNA (*IL-17 siRNA*) and the control siRNA (*Cont siRNA*). Some BMCs were co-cultured with recipient BMMSCs under the stimulation with 1α, 25-(OH)_2_ vitamin D_3_ (*VD3*) and prostaglandin E_2_ (*PGE2*) in the presence of anti-mouse IL-17 antibody (*Anti-IL-17 Ab*) or control antibody for anti-mouse IL-17 antibody (*Cont Ab*). **b** Expression of IL-17 in BMC-MRL/lpr pretreated with IL-17 siRNA and Cont siRNA by Western blotting. **c**, **d** Osteoclast induction assay by tartrate-resistant acid phosphate (*TRAP*) staining. **c** Effects of IL-17 siRNA. **d** Effects of Anti-IL-17 Ab. **e**–**g** Direct osteoclast induction assay of recipient BMCs. **e** A schema of a direct osteoclast induction in a receptor activator for nuclear factor κB ligand and macrophage-colony stimulating factor (*RANKL + M-CSF*) system. Recipient BMCs were pretreated with or without IL-17 siRNA and Cont siRNA and cultured under a stimulation of M-CSF and RANKL. Some BMCs were cultured in the presence of Anti-IL-17 Ab or Cont Ab. **f**, **g **Osteoclast induction assay by TRAP staining. **f** Effects of IL-17 siRNA. **g** Effects of Anti-IL-17 Ab. n = 5 for all groups. Values are shown as means ± SD. *P < 0.05. **c** ^¶^ *P* < 0.05, ^¶¶¶^ *P* < 0.005, versus co-culture of Cont siRNA-treated BMC-MRL/lpr and MSC-MRL/lpr. ^#^ *P* < 0.05, versus co-culture of IL-17 siRNA-treated BMC-MRL/lpr and MSC-MRL/lpr co-culture. **d** ^¶^ *P* < 0.05, ^¶¶^ *P* < 0.01, ^¶¶¶^ *P* < 0.005, versus co-culture of BMC-MRL/lpr and MSC-WT in the presence of Cont Ab. ^#^ *P* < 0.05, versus co-culture of BMC-MRL/lpr and MSC-MRL/lpr in the presence of Anti-IL-17 Ab. *MNC* multinucleated cell; *MSC* mesenchymal stem cell; *SHED* stem cells from exfoliated deciduous teeth

We examined whether IL-17 levels in recipient BMCs of MRL/*lpr* mice play a significant role in osteoclast differentiation through recipient BMMSCs. BMC-MRL/*lpr* were treated with mouse IL-17 siRNA or the control siRNA, and were co-cultured with recipient BMMSCs under stimulation with 1α, 25-(OH)_2_ vitamin D_3_ and prostaglandin E_2_ (Fig. 5a). Some co-cultures were incubated in the presence of anti-mouse IL-17 antibody or the control antibody (Fig. 5a). Western blotting confirmed that siRNA for mouse IL-17 significantly downregulated the expression of IL-17 in BMC-MRL/*lpr* (Fig. 5b). IL-17 siRNA-treated BMC-MRL/*lpr* inhibited individual recipient BMMSC-mediated osteoclast induction in the co-culture system, but control siRNA-treated BMC-MRL/*lpr* did not (Fig. 5c). IL-17 antibody treatment also suppressed osteoclast differentiation in a co-culture system of intact BMC-MRL/*lpr* and individual recipient BMMSCs (Fig. 5d). Furthermore, to understand whether IL-17 levels in recipient BMCs affect direct capabilities of osteoclast differentiation of the BMCs, recipient BMCs were cultured in a RANKL-M-CSF culture system (Fig. 5e). Although IL-17 siRNA treatment in individual BMCs efficiently induced TRAP-positive MNCs, the efficiency was similar to the control cultures treated with control siRNA (Fig. 5f). Anti-IL-17 antibody treatment showed no effect on TRAP-positive MNC formation from individual recipient BMCs in a RANKL-M-CSF culture system when compared to the control antibody treatment (Fig. 5g). In addition, osteoclast formation in a RANKL-M-CSF system expressed a similar efficacy among individual recipient BMCs (Fig. 5f and g). These finding suggested that IL-17 levels in recipient BMCs might affect recipient BMMSC-mediated osteoclast differentiation of recipient BMCs, but not direct osteoclastogenesis of recipient BMCs.

### Systemic treatment of anti-IL-17 antibody improves secondary bone loss in MRL/*lpr* mice

To investigate a role of IL-17 on skeletal metabolism in MRL/*lpr* mice, we systemically infused anti-IL-17 antibody to MRL/*lpr* mice at 16 weeks of age for 4 weeks (Fig. S9a in Additional file 2). MicroCT analyses showed that systemic treatment of anti-IL-17 antibody recovered the BMD and trabecular bone structures when compared to non-infused and isotype-matched IgG2a-infused MRL/*lpr* mice (Fig. S9b-d in Additional file 2). Real-time RT-PCR demonstrated that the long bones of anti-IL-17 antibody-treated MRL/*lpr* mice expressed higher levels of osteoblast-specific genes (*Runx2*, *Alp*, and *osteocalcin*) than those in non-infused and isotype-matched IgG2a-infused MRL/*lpr* mice (Fig. S10a in Additional file 2). Moreover, compared with non-infused and isotype-matched IgG2a-infused MRL/*lpr* mice, anti-IL-17 antibody treatment suppressed expression of osteoclast-specific genes (*Nfatc1, calcitonin receptor*, and *cathepsin K*) by real-time RT-PCR assay (Fig. S10b in Additional file 2). ELISA also demonstrated that anti-IL-17 antibody treatment markedly reduced serum bone resorption markers including sRANKL and CTX in comparison with non-infused and isotype-matched IgG2a-infused MRL/lpr mice (Fig. S10c in Additional file 2). These findings suggested that systemic anti-IL-17 antibody treatment might recover the osteoporotic disorder in MRL/*lpr* mice via accelerated bone formation and suppressed bone resorption.

## Discussion

The incidence of secondary osteoporosis in SLE patients ranges from 6.3 % to 28 % worldwide [30, 31]. Osteoporosis is generally caused by disruption of bone remodeling, which is related to menopause and aging, whereas secondary osteoporosis in SLE is multifactorial and involves excessive systemic inflammation and long-term anti-inflammatory/immunosuppressive medications such as glucocorticoids [25]. These risk factors lead to fragility fractures, which are mainly vertebral fractures, and can proceed to new fractures, increase the mortality rate, and reduce quality of life [32, 33]. Among the numerous anti-osteoporosis drugs to prevent and treat bone loss in SLE patients, bisphosphonates are a first-line, anti-resorptive agent but show little effect on bone reconstruction and exhibit side effects such as negative fetus development, osteonecrosis of the jaw, and musculoskeletal pain. Other anti-osteoporotic agents, such as estrogen, calcitonin, and raloxifene, exhibit various limitations in terms of potency, population effects, and side effects [34]. From the viewpoint of a quality of life-based medicine, novel therapeutics have been strongly desired to recover the bone loss in SLE patients. In this study, according to recent findings of human MSC-based therapy [14–18], we systemically transplanted hBMMSCs and SHED into SLE model MRL/*lpr* mice with a severe osteoporotic phenotype and assessed their therapeutic effects on bone loss. Our results demonstrated that human MSC-based therapy ameliorated bone reduction in MRL/*lpr* mice.

Upon dysregulation of the bone remodeling balance by osteoblasts and osteoclasts, the skeletal system enters a pathological condition. In bone marrow, BMMSCs not only serve as a source of osteoblasts, but also support osteoclast differentiation [25–27]. Increasing evidence has shown impaired functions in BMMSCs derived from SLE patients [10, 19, 35, 36] and SLE model mice [10], suggesting that a recipient BMMSC deficiency might participate in the pathology of secondary osteoporosis in SLE. However, recent studies demonstrate that systemic MSC transplantation into MRL/*lpr* mice improves the bone loss, although no study has focused on recipient BMMSCs in the bone regeneration process by systemic transplantation of MSCs [10, 16, 17]. Therefore, we hypothesized that the therapeutic effects of systemically transplanted MSCs on bone regeneration in SLE were mediated by recovery of deficient host BMMSCs. Interestingly, in the present study, the exogenous hBMMSCs and SHED recovered the impaired bone-forming capability of recipient BMMSCs and reduced abnormal osteoclast induction via recipient BMMSCs in the bone marrow of MRL/*lpr* mice. These findings suggest that recovery of the impaired recipient BMMSCs might be a critical process to regenerate the skeletal loss in MRL/*lpr* mice after human MSC transplantation. However, further studies should explore the cellular and molecular mechanisms through which exogenous MSCs improve the deficiency of recipient BMMSCs.

Inflammation shifts skeletal homeostasis to a bone resorptive condition. Secondary osteoporosis in SLE involves complex interplay of hyperactivated immune reactions and abnormal bone metabolism. The proinflammatory cytokine IL-17, which is produced by Th17 cells [37], has been investigated as a participant in various autoimmune diseases [38, 39]. These findings suggest a novel role for IL-17 in bone diseases such as rheumatoid arthritis and osteoporosis [40, 41]. In this study, we demonstrated that IL-17-dependent hyperimmune conditions in the recipient bone marrow in MRL/*lpr* mice impaired recipient BMMSCs to suppress the osteogenic function and accelerate the osteoclast induction. Our IL-17 neutralization experiments also provided strong evidence that abnormal IL-17 expression in the bone marrow of MRL/*lpr* mice impaired recipient BMMSC-mediated osteogenesis and recipient BMMSC-mediated osteoclast induction. However, our siRNA and neutralizing antibody of IL-17 experiments also demonstrated that IL-17 levels in recipient BMCs might affect recipient BMMSC-mediated osteoclast differentiation of recipient BMCs, but not influence osteoclastogenesis of recipient BMCs directly. In bone metabolism, IL-17 induces osteoclastic differentiation through osteoclastogenesis-supporting cells, such as mesenchymal cells and osteoblasts, but not in direct osteoclast induction stimulated by M-CSF and RANKL [42, 43]. IL-17 also directly inhibits osteoblastic differentiation of MSCs [44]. Therefore, these findings suggest that hyperactivated IL-17 in recipient bone marrow may be responsible for secondary osteoporosis in MRL/*lpr* mice by impairing recipient BMMSCs. Furthermore, the present systemic transplantation of hBMMSCs and SHED suppressed the increased expression of bone marrow IL-17 in MRL/*lpr* mice, and restored the impaired functions of recipient BMMSCs in bone metabolism. NF-κB activation by proinflammatory cytokines including IL-17 is known to reduce the bone formation capacity of host BMMSCs [19, 44]. Further efforts will be required to elucidate the crucial mechanism of IL-17 in the recipient BMMSC-based pathology of secondary osteoporosis in SLE, which may lead to novel recipient BMMSC-targeting therapeutics for skeletal disorders.

IL-17 antibody treatments have emerged as a novel therapeutic approach for immune-mediated diseases such as psoriasis, rheumatoid arthritis, psoriatic arthritis and ankylosing spondylitis [45, 46]. Experimental evidence demonstrated anti-IL-17 therapy could protect bone destruction in rheumatoid arthritis by reducing the number of osteoclasts in joints as well as Th17 cells [47]. Anti-IL-17 antibody also preserves skeletal loss in osteoporosis by enhancing osteoblastic bone formation and suppressing osteoclastic bone resorption, as well as protecting against IL-17-mediated immune damages [48]. Several direct IL-17 inhibitors (for example, secukinumab and ixekizumab, anti-IL-17A monoclonal antibody) have shown exciting advances in proof-of-concept and phase II clinical trials, but also expected are further evaluations from phase III clinical trials in multiple autoimmune and immune-related inflammatory diseases [49]. In this study, we demonstrated that systemic treatment of anti-IL-17 antibody recovered bone reduction in MRL/*lpr* mice. The usage of IL-17 antibody in treating secondary osteoporosis will not only provide a therapeutic method but also improve the understanding of disease pathogenesis.

In this study, we found that pathological immune conditions affected recipient MSCs, and indicated that hBMMSCs and SHED transplantation improved the bone reduction by restoring IL-17-impaired recipient BMMSCs after migration into the damaged bone marrow. These results suggest that abnormal recipient BMMSCs may undergo correction of their primary functions by the post-transplantation actions of human MSCs. Although the therapeutic mechanism of engrafted human MSCs in the target bone site is not fully understood, several possibilities may be involved in the post-transplantation behaviors of human MSCs in impaired bone marrow. Migrated human MSCs have the potential to participate directly in bone regeneration by differentiating into osteoblasts and suppressing osteoclast induction [25–27]. However, bone reconstruction is affected by proinflammatory cytokines at bone defect sites [50]. In addition, MSCs act as cellular modulators through immunomodulatory and trophic effects [51]. Immunomodulatory functions are inducted by cell-cell contact and include FasL-mediated T cell apoptosis [52], CCR6-mediated Th17 cell inhibition [53], and MSC-secreted molecule (e.g., IL-10)-mediated Th17 cell suppression [54]. Trophic molecules released from MSCs can inhibit apoptosis and scar formation, such as macrophage inflammatory protein-1, stromal cell-derived factor-1, transforming growth factor-β1, and vascular endothelial growth factor [55]. Therefore, exogenous MSCs may exert their immunomodulatory and trophic functions by secreting bioactive molecules to recover impaired recipient BMMSCs. Further studies will be necessary to explore the deeper mechanisms of transplanted MSC-mediated recovery of recipient BMMSCs.

## Conclusions

The present study demonstrates that systemic transplantation of human MSCs, including SHED and hBMMSCs, ameliorates severe bone reduction, as well as primary SLE disorders, in MRL/*lpr* mice. The therapeutic efficacy is mediated by recovery of the impaired functions of recipient BMMSCs to regulate osteogenesis and osteoclastogenesis via IL-17 suppression in the recipient bone marrow. These data indicate that IL-17, as a cause of secondary osteoporosis in SLE, might be a therapeutic target of transplanted human MSCs in the skeletal disorder. Further studies will be necessary to explore new cellular and molecular strategies to overcome the recipient BMMSC-based pathology of secondary osteoporosis in SLE and develop a novel recipient BMMSC-targeting approach in MSC-based therapy for skeletal regeneration.

## Additional files

Additional file 1: **Supplementary materials and methods.**

Additional file 2: **Supplementary Figs. 1-10. **

## Abbreviations

ALP: alkaline phosphatase
αMEM: alpha minimum essential medium
BMC: bone marrow cell
BMC-WT: wild-type mice-derived bone marrow cells
BMD: bone mineral density
BMMSC: bone marrow mesenchymal stem cell
BV/TV: bone volume/trabecular volume
Calvaria-WT: wild-type mouse-derived calvarial cells
CFSE: carboxyfluorescein diacetate succinimidyl ester
CFU-F: colony-forming unit fibroblasts
CM: conditioned medium
CTX: C-terminal telopeptides of type I collagen
ELISA: enzyme-linked immunosorbent assay
FBS: fetal bovine serum
hBMMSC: human bone marrow mesenchymal stem cell
hUCMSC: human umbilical cord-derived mesenchymal stem cell
IFN: interferon
IL: interleukin
M-CSF: macrophage-colony stimulating factor
microCT: micro-computed tomography
MNC: multinucleated cell
MSC: mesenchymal stem cell
MSC-WT: wild-type mice-derived mesenchymal stem cells
NFATc1: nuclear factor of activated T-cells
NF-κB: nuclear factor κB
PBS: phosphate-buffered saline
RT-PCR: reverse transcription polymerase chain reaction
Runx2: runt-related transcription factor 2
SHED: stem cells from exfoliated deciduous teeth
SLE: systemic lupus erythematosus
sRANKL: soluble receptor activator for nuclear factor κB ligand
Tb.N: trabecular number
Tb.Sp: trabecular separation
Tb.Spac: trabecular space
Tb.Th: trabecular thickness
Th17: IL-17 producing helper T
TRAP: tartrate-resistant acid phosphate
