# Supplementary material for: Transplantation of mesenchymal stem cells ameliorates secondary osteoporosis through interleukin-17-impaired functions of recipient bone marrow mesenchymal stem cells in MRL/lpr mice
Source: Stem Cell Res Ther. 2015 May 27;6(1):104. doi: 10.1186/s13287-015-0091-4 (PMC4474573; doi:10.1186/s13287-015-0091-4)
Supplement: Supplementary file 1 — Supplementary materials and methods. [file 13287_2015_91_MOESM1_ESM.pdf]

Supplementary Materials and Methods

Systemic MSC transplantation into C57BL/6 mice

P3 hBMMSCs and SHED diluted in PBS were intravenously infused at 1×10^5 per 10 g of body weight into 16-week-old C57BL/6 mice (CLEA Japan, Tokyo, Japan) via the right cervical vein according to a previously published method [1 in Supplementary References in Additional file 1] (Figure S1A in Additional file 2). PBS-infused mice were used as the controls. The mice were analyzed at 20 weeks of age. Age-matched MRL/*lpr* mice that received PBS were used as controls. All animal experiments were approved by the Institutional Animal Care and Use Committee of Kyushu University (protocol number: A21-044-1).

Biochemical assay of biological (blood serum and urines) and culture (culture supernatant) samples

Albumin, anti-double strand DNA (dsDNA) IgG and IgM antibodies and anti-nuclear antigen (ANA) in biological samples were measured by using enzyme linked immunosorbent assay (ELISA) kits (Albumin: R&D Systems, Minneapolis, MN; anti-dsDNA antibodies and ANA: Alpha Diagnostic, San Antonio, TX). Serum creatinine and urine protein was assayed by using creatinine parameter assay kit (R&D Systems) and Bio-Rad protein assay (Bio-Rad, Hercules, CA), respectively.

Histological bone analysis

Tibiae were fixed with 4% paraformaldehyde in PBS and decalcified with 10% ethylenediaminetetraacetic acid. Paraffin sections were prepared at a thickness of 6 μm. The

sections were treated with hematoxylin and eosin [2 in Supplementary References in Additional file 1]. For immunofluorescence, frozen sections were prepared at a thickness of 6 μm. The cryosections were treated with non-immune IgG and reacted with an anti-mouse IL-17 antibody (Santa Cruz Biotechnology, Dallas, TX) or the isotype-matched antibody (Santa Cruz Biotechnology). The sections were then stained with CF 633-conjugated secondary antibody (Biotium, Hayward, CA) and 4', 6-diamidino-2-phenylindole (DAPI) (Dojindo, Kumamoto, Japan).

In vivo tracing of human MSCs

hBMMSCs and SHED (1×10^7 each) were incubated with 10 μg carboxyfluorescein diacetate succinimidyl ester (CFSE) (Invitrogen, Carlsbad, CA) in PBS for 10 minutes at 37°C. The CFSE-labeled hMSCs were intravenously infused at 1×10^6 per mouse into 16-week-old MRL/*lpr* mice. At 1 or 7 days after infusion, long bones were fixed with 4% PFA in PBS and decalcified with 10% EDTA. Frozen sections were then prepared and stained with DAPI (Dojindo).

Colony forming unit fibroblastic assay

Recipient bone marrow cells (BMCs) (1.5×10^6/flask) were seeded in T-25 flasks. After 3 hours, the cells were washed with PBS and cultured for 16 days. Then, the cells were treated with a 2% PFA and 1% toluidine blue solution. Cell clusters containing ≥50 cells were recognized as a colony under a light microscope. Total colony numbers were counted per flask.

Population doubling assay

Recipient BMMSCs were isolated based on the CFU-F method (Yamaza et al., 2008). They were seeded in T-75 culture flasks, and were cultured to sub-confluency and then passaged. These steps were repeated until senescence. The population-doubling score was calculated at every passage according to the equation: $\log_2$ (number of final harvested cells/number of initial seeded cells) and determined by the total score.

Proliferation assay of mouse BMMSCs

Recipient BMMSCs (1×10^4/well) were seeded in 8-well chamber slides, incubated with a bromodeoxyuridine (BrdU) solution (1:100) (Invitrogen) for 20 hours, and then stained using a BrdU staining kit (Invitrogen). BrdU-positive cell numbers were calculated as a percentage of the total cell number in 10 images per subject.

In vitro osteogenic capacity of mouse BMMSCs

Recipient BMMSCs isolated from wild-type C57BL/6J mice were cultured under the osteogenic condition. The osteogenic medium consisted of 20% fetal bovine serum (FBS) (Equitech-Bio, Kerrville, TX), 2 mM L-glutamine (Nacalai Tasque, Kyoto, Japan), 55 μM 2-mercaptoethanol (Invitrogen, Carlsbad, CA), 100 mM L-ascorbic acid 2-phosphate (Wako Pure Chemical Industrial, Osaka, Japan), 2 mM β-glycerophosphate (Sigma, St. Louis, MO), 10 nM dexamethasone (Sigma) and mixed antibiotics including 100 U/ml penicillin and 100 mg/ml streptomycin (Nacalai Tesque) in alpha Modification of Eagle's Medium (αMEM) (Invitrogen) with or without conditioned medium (CM) of mouse bone marrow cells (BMCs), 10 nM recombinant mouse interleukin 17 (IL-17) (R&D Systems) and 1 mg/mL anti-mouse IL-17

antibody (R&D Systems). Four weeks after the induction, Alizarin Red-positive area was quantified by using NIH Image-J.

In vitro osteoclast assay

Mouse BMCs (1×10^6/well) isolated from wild-type C57BL/6J mice were co-cultured for 7 days with mouse calvarial cells (0.1×10^6/well) pretreated with or without CM of mouse BMCs, 10 nM recombinant mouse IL-17 and 1 μg/mL anti-mouse interleukin IL-17 antibody for 3 days. The CM that enriched tenfold was mixed with the growth medium at the ratio of 1:9. Mouse calvarial cells were isolated from 2-3 day-old wild type C53BL/6 mice with a sequential enzyme treatment [3 in Supplementary References in Additional file 1]. The osteoclastogeic medium contained 10% FBS, 100 U/ml penicillin and 100 mg/ml streptomycin, 10 nM vitamin D_3 (Wako Pure Chemical Industrial, Osaka, Japan) and 1 nM prostagrandine E_2 (Wako Pure Chemical Industrial) in αMEM. TRAP-positive multinucleated cells (>3 nuclei) were determined as osteoclast-like cells.

Quantitative real-time polymerase chain reaction assay for osteoblast- and osteoclast-specific gene expression

Total RNAs were extracted from samples with TRIzol (Invitrogen), digested with DNase I (Promega, Madison, WI) and purified using an RNeasy Mini Kit (Qiagen). One microgram of purified RNA was reverse-transcribed with a Revertra Ace qPCR kit (TOYOBO). Real-time PCR was subsequently performed using a TaqMan Gene Expression Master Mix (Applied Biosystems, Foster City, CA) and target TaqMan probes as follows; mouse alkaline phosphatase (Mm00475834_m1; Applied Biosystems), calcitonin receptor (Mm00432271_m1; Applied

Biosystems), cathepsin K (Mm00484039_m1; Applied Biosystems), *nuclear factor of activated T-cells* (Mm00479445_m1; Applied Biosystems), *runt-related transcription factor 2* (Mm00501584_m1; Applied Biosystems), and *osteocalcin* (sense primer, 5-GCAATAAGGTAGTGAACAGACTCC-3; antisense primer, 5-GTTTGTAGGCGGTCTTCAAGC-3; probe, 5-TGGAGCCTCAGTCCCCAGCCCA-3) genes and normalized it to the expression of the housekeeping gene *glyceraldehyde 3-phosphate dehydrogenase* (Mm99999915_g1; Applied Biosystems),.

Induction assay of human interleukin 17 (IL-17)-producing helper T (Th17 cells) cells.

Human $CD4^{+}CD25^{-}$ naïve T lymphocytes (1×10^{6} per well) were magnetically sorted from human PBMNCs (AllCells, Barkley, CA) using $CD4^{+}CD25^{+}$ regulatory T cell isolation kit (Miltenyi Biotec, Auburn, CA) and activated by plate-bounded anti-CD3 (5 μg/ml) (eBioscience, San Diego, CA) and soluble anti-CD28 (1 μg/ml) (eBioscience) antibodies for 3 days. The activated T cells were loaded on the MSC cultures (20×10^{3} per well) with human transforming growth factor b_1 (TGF-β_1) (2 μg/ml) (R&D Systems) and interleukin 6 (IL-6) (50 μg/ml) (R&D Systems) for 3.5 days. Floating cells ($1x10^{6}$) were incubated with PerCP-conjugated anti-CD4, FITC-conjugated anti-CD8a, followed by the treatment with R-PE-conjugated anti-IL-17 and APC-conjugated anti-interferon gamma (IFNγ) antibodies using a Foxp3 staining buffer kit (eBioscience) as previously, and then analyzed $CD4^{+}IL17^{+}IFN\gamma^{-}$ cells as Th17 cells on FACSVerse flow cytometer (BD Biosciences, San Jones, CA).

Assay for mouse peripheral Th17 cells

Mouse peripheral blood cells (0.1×10^6 per 100 ml) were incubated with PerCP-conjugated anti-CD4 antibody (1 μg) (eBioscience) and treated with R-PE-conjugated anti-IL-17 (eBioscience) and APC-conjugated anti-IFNγ (eBioscience) antibodies (each 1 μg) by using Fixation/permiabilization kit (eBioscience). As controls, the isotype-matched antibodies (eBioscience) were used. Finally the number (percentage) of $CD4^+IL\text{-}17^+IFN\gamma^-$ Th17 cells (per 10×10^3) was measured on FACSVerse (BD Biosciences).

Anti-IL-17 antibody treatment

We intraperitoneally injected rat anti-mouse interleukin-17 (IL-17) IgG2a antibody (R&D Systems) (100 μg/100 μl in PBS) or the isotype-matched rat IgG2a (100 μg/100 μl in PBS) (R&D Systems) twice a week into 16-week-old MRL/*lpr* mice (Figure S9A in Additional file 1). PBS-injected mice were used as non-injected group. After the four-week-treatment, the animals were sacrificed to harvest peripheral blood and femurs.

Microcomputed tomographic bone analysis

Femoral bones of mice were analyzed by microcomputed tomography (microCT) with a 1076 microCT system (Skyscan, Kontich, Belgium), as previously described [3 in Supplementary References in Additional file 1]. Density values were calibrated using hydroxyl apatite phantoms with bone mineral density [BMD] values of 0.25 and 0.75 g/cm^3 (Skyscan). BMD and bone structural indices (bone volume/trabecular volume [BV/TV], trabecular thickness [Tb.Th], trabecular number [Tb.N], and trabecular separation [Tb.Sp]) were calculated.

Statistical analysis

Data were assayed by a one-way ANOVA F test. Values of $P < 0.05$ were considered to be significant.

Supplementary References

1. Yamaza T, Akiyama K, Chen C, Liu Y, Shi Y, Gronthos S, Wang S, Shi S: **Immunomodulatory properties of stem cells from human exfoliated deciduous teeth.** *Stem Cell Res Ther* 2010, **1**:5.
2. Yamaza T, Miura Y, Akiyama K, Bi Y, Sonoyama W, Gronthos S, Chen W, Le A, Shi S: **Mesenchymal Stem Cell-Mediated Ectopic Hematopoiesis Alleviates Aging-Related Phenotype in Immunocompromised Mice.** *Blood* 2009, **113**:2595-2604.
3. Yamaza T, Miura Y, Bi Y, Liu Y, Akiyama K, Sonoyama W, Patel V, Gutkind S, Young M, Gronthos S, Le A, Wang CY, Chen W, Shi S: **Pharmacologic stem cell based intervention as a new approach to osteoporosis treatment in rodents.** *PLoS One* 2008, **3**:e2615.
