# Supplementary material for: Transplantation of mesenchymal stem cells ameliorates secondary osteoporosis through interleukin-17-impaired functions of recipient bone marrow mesenchymal stem cells in MRL/lpr mice
Source: Stem Cell Res Ther. 2015 May 27;6(1):104. doi: 10.1186/s13287-015-0091-4 (PMC4474573; doi:10.1186/s13287-015-0091-4)
Supplement: Supplementary file 2 — Supplementary Figs. 1-10. [file 13287_2015_91_MOESM2_ESM.pdf]

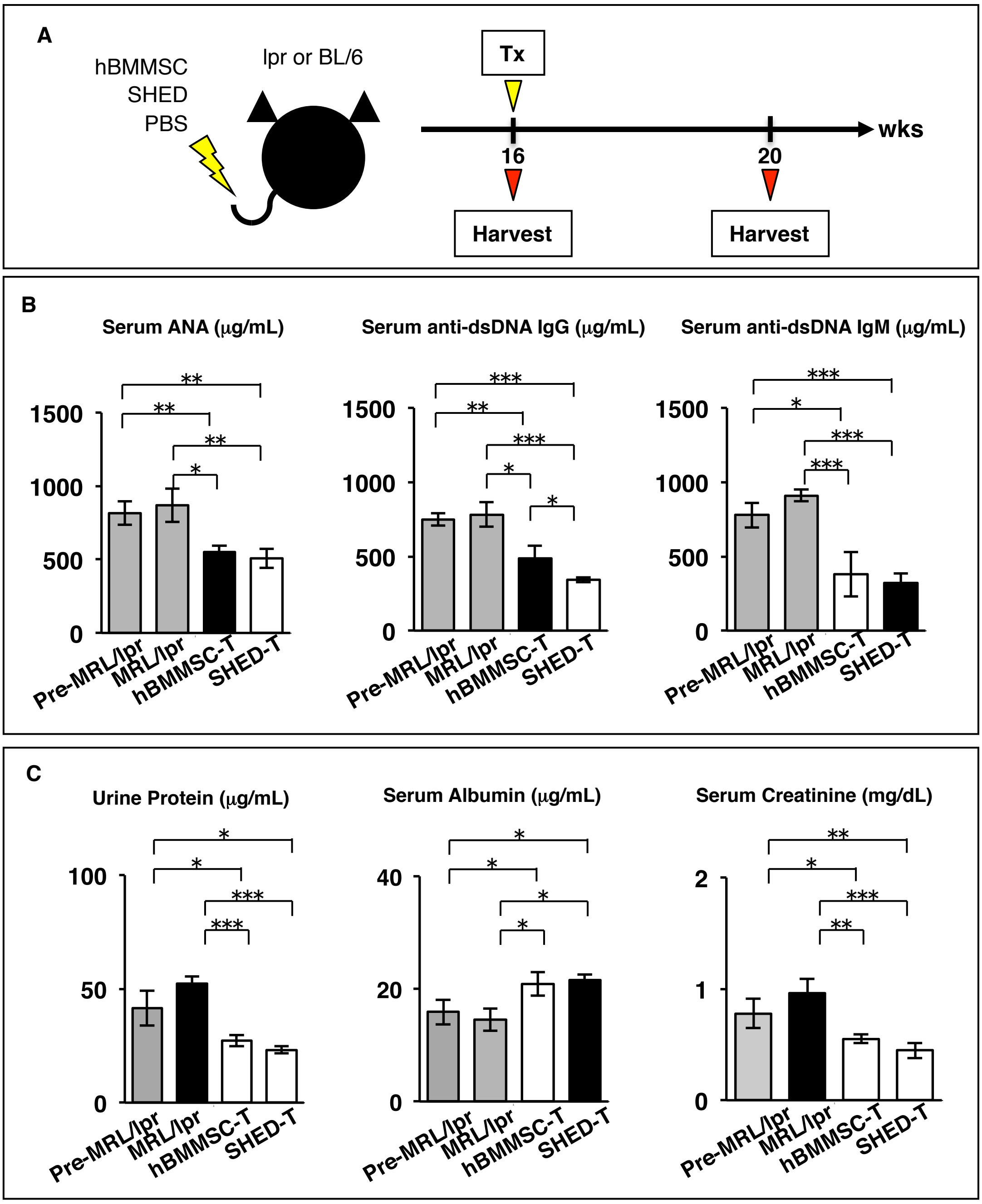

Figure S1. **Systemic transplantation of hMSCs improves serum hyper-autoantibody levels and renal dysfunction in MRL/*lpr* mice.** (**A**) A schema of systemic transplantation (Tx) of hBMMSCs and SHED into MRL/*lpr* (lpr) or C57BL/6 (BL/6) mice. The mice were recieved the transplants at 16 weeks (wks) of age. The control mice were treated with PBS. (**B**) ELISA of serum autoantibody. ANA: anti-nuclear antibody. (**C**) Levels of urine protein and serum albumin and creatinine. **B, C:** n=5 for all group. Pre-MRL/lpr: pre-transplant MRL/*lpr* mice at 16 weeks of age; MRL/lpr, hBMMSC-T, SHED-T: non-, hBMMSC-, and SHED-transplanted MRL/*lpr* mice at 20 weeks of age, respectively. **P*<0.05, ***P*<0.01, and ****P*<0.005. The graph bars represent means±SD.

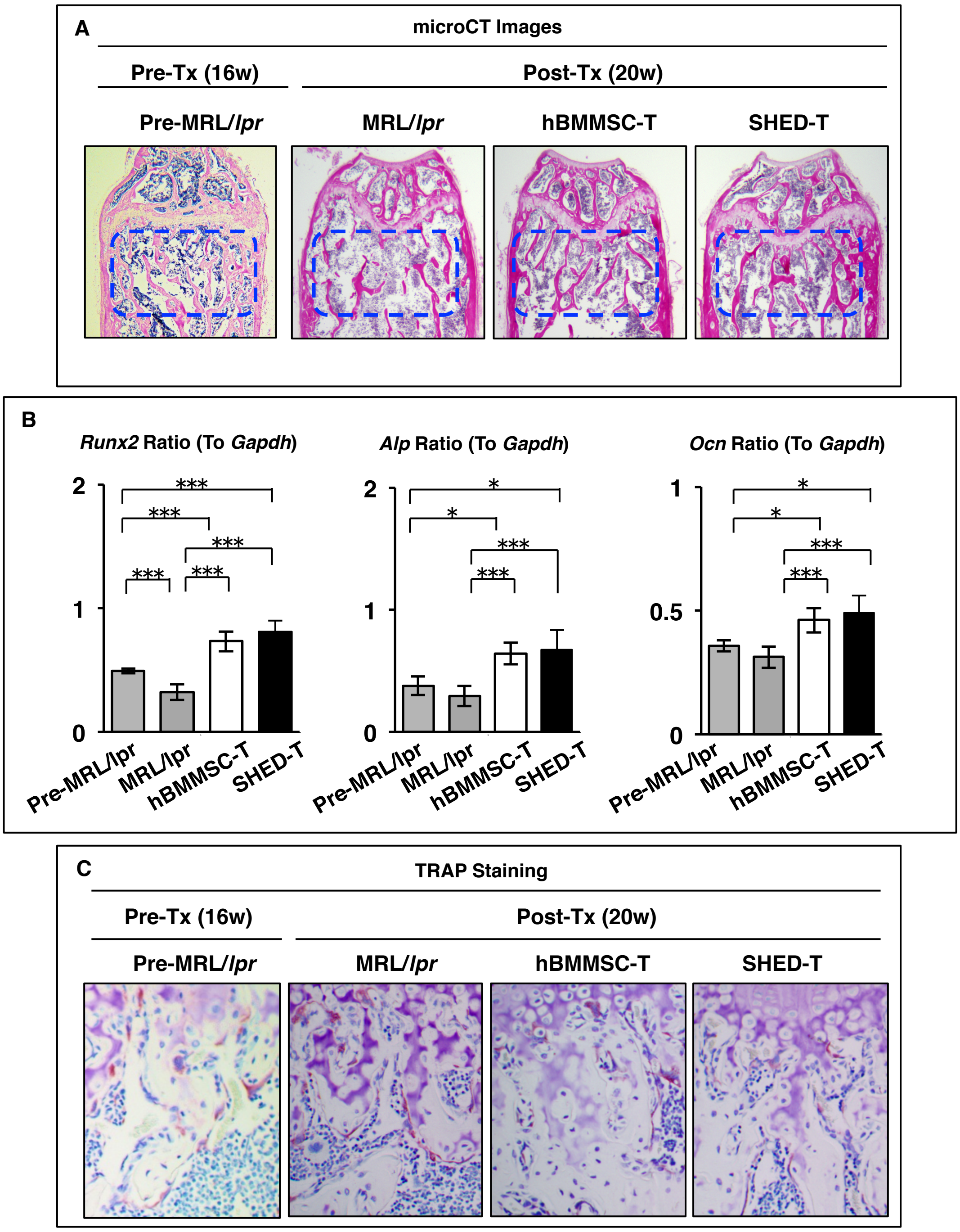

Figure S2. Systemic hMSC transplantation ameliorates the bone loss in MRL/*lpr* mice. (A) Representative histological images of trabecular bone structures (blue-dashed areas). (**B**) *In vivo* osteoblast activity assay. Real-time RT-PCR analysis of osteoblast-specific genes. *Alp*: alkaline phosphatase; *Gapdh*: Glyceraldehyde 3-phosphate dehydrogenase; *Ocn*: osteocalcin; *Runx2*: runt-related transcription factor 2. (**C**) *In vivo* osteoclast activity assay. TRAP staining of recipient tibias. **A–C:** n=5 for all groups. Pre-Tx (16w): pre-transplant stage at 16 weeks of mouse age; Post-Tx (20w): post-transplanted stage at 20 weeks of mouse age. **B:** *P<0.05 and ***P<0.005. Bar graphs show the means ±SD.

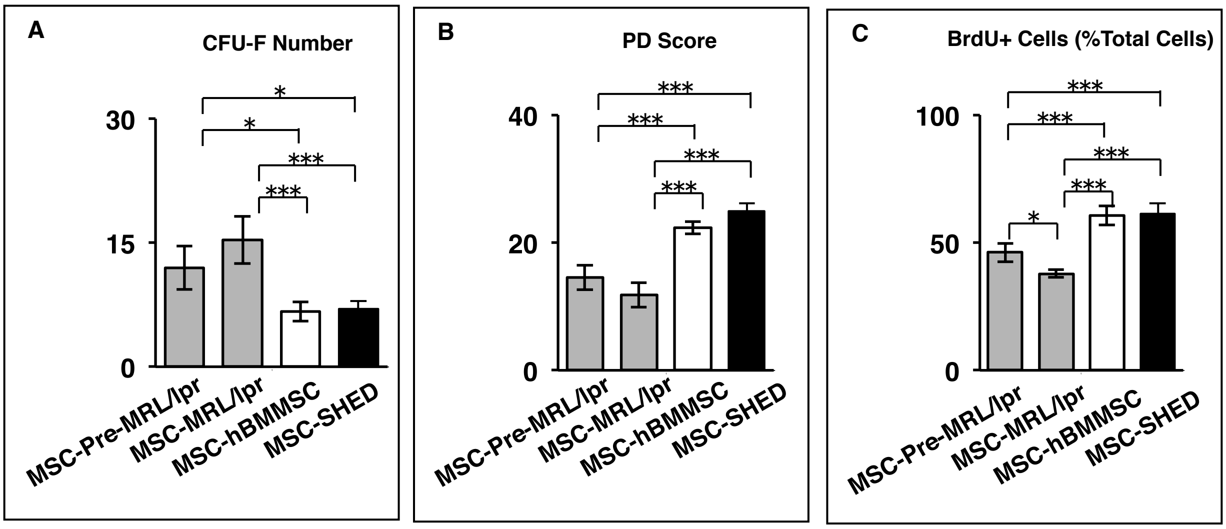

Figure S3. Systemic hMSC transplantation recovers dysregulation of recipient BMMSCs in MRL/*lpr* mice. (**A**–**C**) Stemness of recipient BMMSCs. Colony forming unit-fibroblasts (CFU-F) assay (**A**). Population doubling (PD) assay (**B**). Brd-U incorporation assay (**C**). **A**–**C:** n=5 for all groups. *P<0.05 and ***P<0.005. Bar graphs show the means±SD. MSC-Pre-MRL/lpr, MSC-MRL/*lpr*, MSC-hBMMSC, MSC-SHED: BMMSCs isolated from pre-transplant MRL/*lpr* mice at 16 weeks of age, and non-, hBMMSC-, and SHED-transplanted MRL/*lpr* mice at 20 weeks of age, respectively.

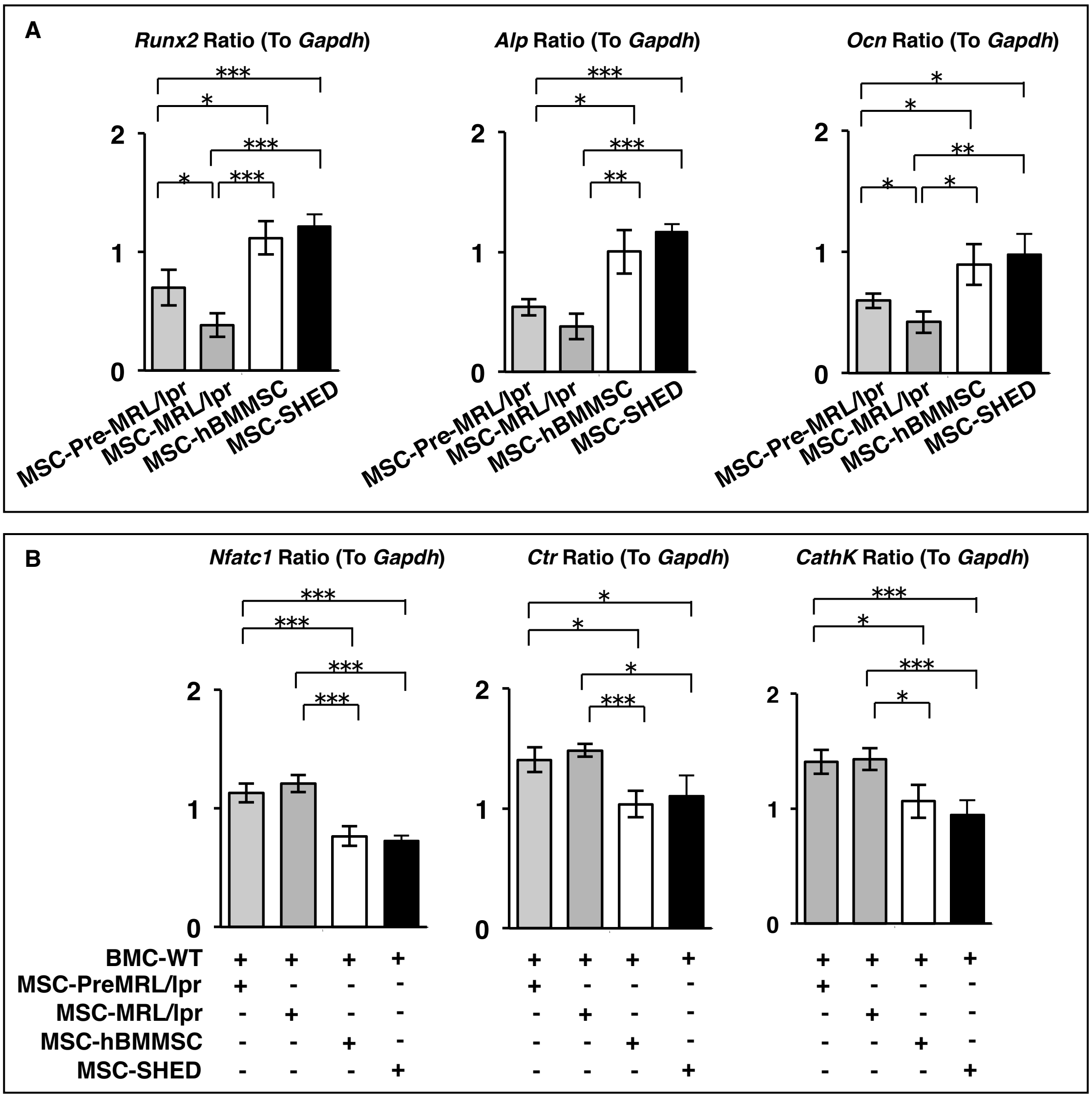

Figure S4. Systemic hMSC transplantation recovers dysregulation of osteoblastogenesis and osteoclastogenesis via recipient BMMSCs in MRL/*lpr* mice. (**A**) Osteogenic activity of recipient BMMSCs. Real-time RT-PCR analysis of osteoblast-specific genes at 1 week after osteogenic induction. (**B**) Osteoclast inductivity of recipient BMMSCs in co-culture with wild-type C57BL/6 mice-derived bone marrow cells (BMC-WT) under stimulation with 1a, 25-$(OH)_2$ vitamin D_3 and prostaglandin E_2 for 7 days . Real-time RT-PCR analysis of osteoclast-specific gene assay. *Nfatc1*: nuclear factor of activated T cells, cytoplasmic 1; *Ctr*: calcitonin receptor, *CathK*: cathepsin K; *Gapdh*: glyceraldehyde 3-phosphate dehydrogenase. **A, B:** n=5 for all groups. MSC-Pre-MRL/*lpr,* MSC-MRL/*lpr*, MSC-hBMMSC, MSC-SHED: BMMSCs isolated from pre-transplant, non-, hBMMSC-, and SHED-transplanted MRL/*lpr* mice, respectively. **P*<0.05, ***P*<0.01, and ****P*<0.005. Bar graphs show the means±SD.

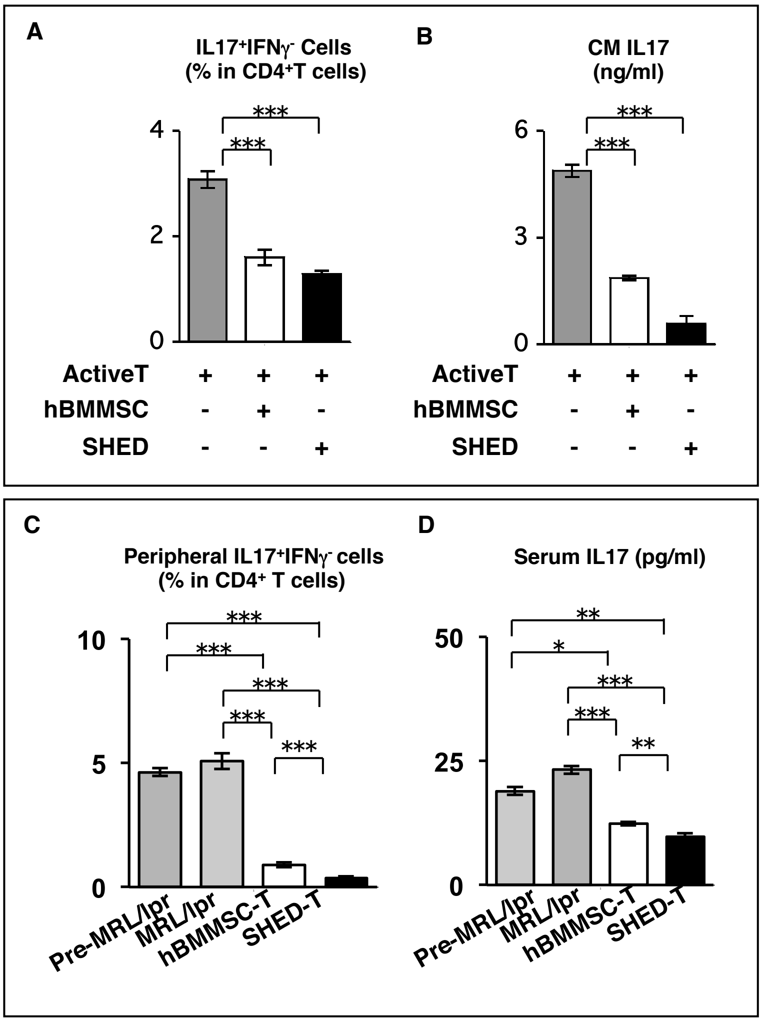

Figure S5. Systemic transplantation of hMSCs suppresses peripheral Th17 cells in MRL/lpr mice. (**A, B**) Suppression of Th17 cell differentiation and IL-17 secretion by hBMMSCs and SHED in co-culture. Flow cytometry of $CD4^{+}IL\text{-}17^{+}IFN\gamma^{-}$ cells (**A**). ELISA of IL-17 in conditioned medium (CM IL17) (**B**). (**C, D**) Peripheral levels of Th17 cells and IL-17 in recipient MRL/*lpr* mice. Flow cytometry of peripheral $CD4^{+}IL\text{-}17^{+}IFN\gamma^{-}$ cells (**C**). ELISA of serum IL-17 (serum IL17) (**D**). *P<0.05, **P<0.01, and ***P<0.005. The graph bar represents means±SD. n=5 for all groups. **A, B**: Active T: human $CD4^{+}CD25^{-}$ T cells activated with anti-CD3 and anti-CD28 antibodies.

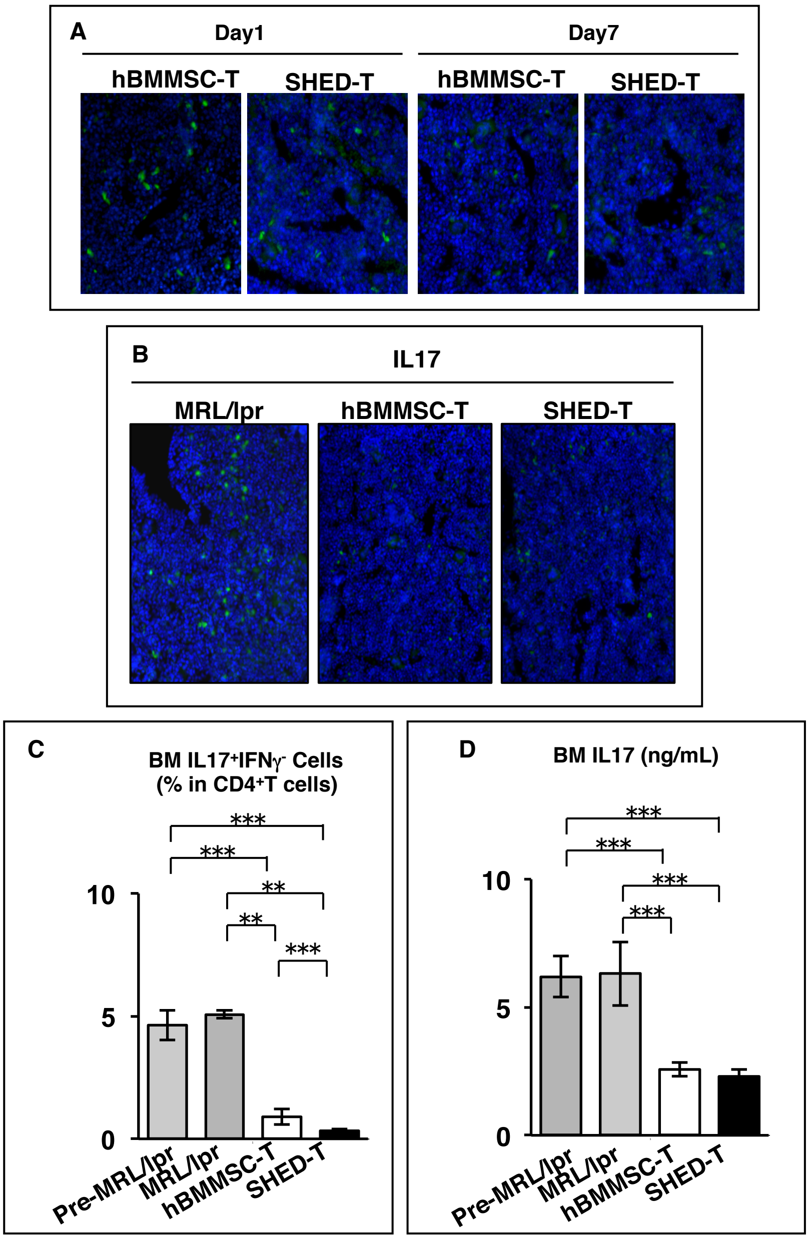

Figure S6. Systemic hMSC transplantation suppresses the IL-17-enhanced environment in recipient bone marrow of MRL/*lpr* mice. (**A**) *In vivo* homing assay of hBMMSCs and SHED in recipient bone marrow. Cell tracing assay using the CSFE-labeling method. Nuclei were counterstained with DAPI. Day 1: Cell infusion after 1 day; Day 7: Cell infusion after 7 days. (**B–D**) Levels of IL-17 and Th17 cells in recipient bone marrow. Immunofluorescence of IL-17 (IL17). Nuclei were counterstained with DAPI. (**B**). Flow cytometric analysis of $CD4^+IL\text{-}17^+IFN\gamma^-$ cells in bone marrow (BM $CD4^+IL\text{-}17^+IFN\gamma^-$ cells) (**C**). ELISA of bone morrow IL-17 (BM IL17) (**D**). **A–D:** n=5 for all groups. **C, D:** $^{**}P<0.01$ and $^{***}P<0.005$. Bar graphs show the means±SD.

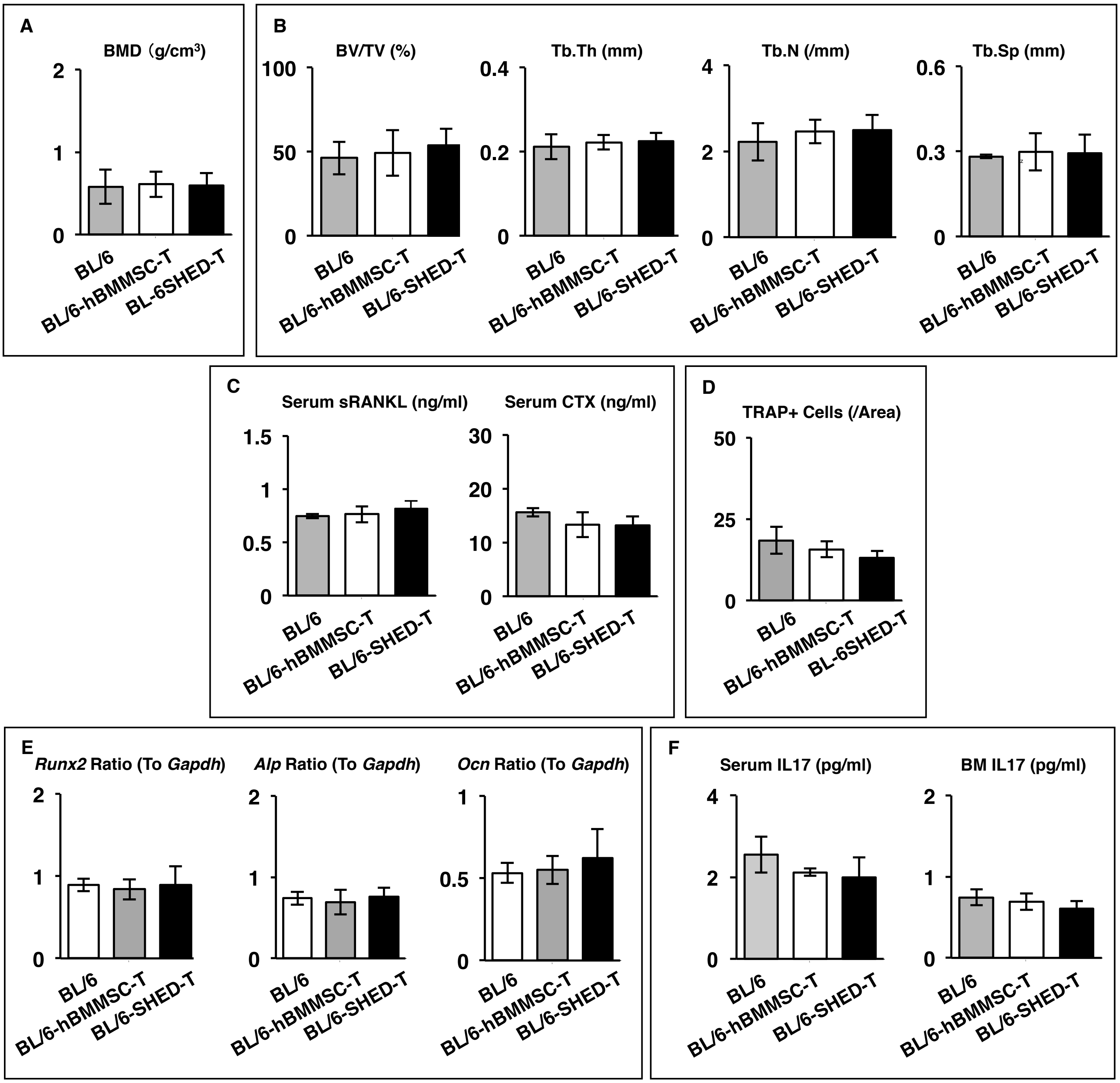

Figure S7. Systemic hMSC transplantation shows no effect to the bone loss and IL-17 levels in C57BL/6 mice. (A, B) Morphological analyses of mouse tibiae. Bone mineral density (BMD) (**A**). Trabecular bone parameter assay. BV/TV: bone volume ratio to tissue volume; Tb.Th: trabecular thickness; Tb.N: trabecular number; Tb.Sp: trabecular separation (**B**). (**C**, **D**) *In vivo* osteoclast activity assay. ELISA of mouse serum. CTX: C-terminal telopeptides of type I collagen; sRANKL: soluble RANKL (**C**). Histological analysis of recipient tibias by TRAP staining. TRAP+ cells: TRAP-positive osteoclast-like cells (**D**). (**E**) In vivo osteoblast activity assay. Real-time RT-PCR analysis of osteoblast-specific genes. *Alp*: alkaline phosphatase; *Gapdh*: Glyceraldehyde 3-phosphate dehydrogenase; *Ocn*: osteocalcin; *Runx2*: runt-related transcription factor 2. (**F**) IL-17 levels in serum and bone marrow. ELISA. **A–F:** n=5 for all groups. BL/6, BL/6-hBMMSC-T, BL/6-SHED-T: non-, hBMMSC-, and SHED-transplanted C57BL/6 mice. Bar graphs show the means±SD.

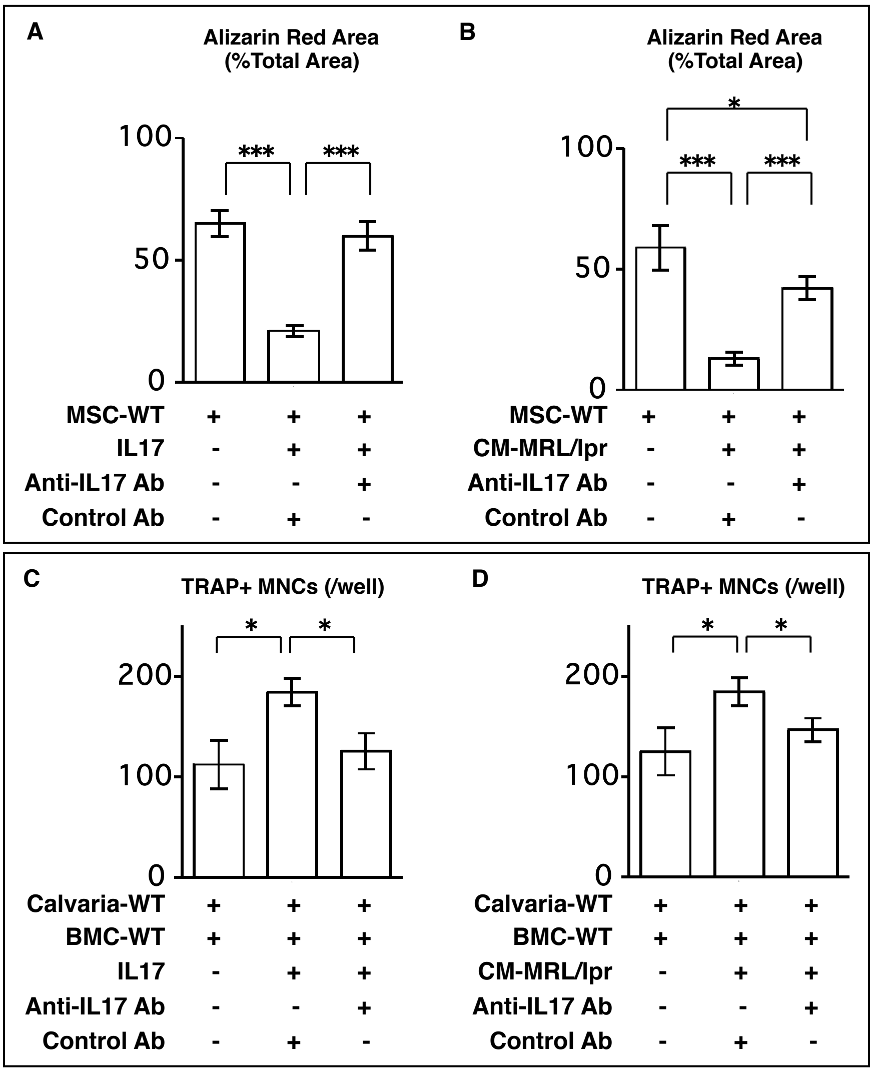

Figure S8. Effects of IL-17 on osteogenic capacity and osteoclast differentiation. (**A, B**) Alizarin Red staining 4 weeks after the osteogenic induction of wild type mouse-derived BMMSCs (MSC-WT) under recombinant mouse IL-17 (IL17) (**A**) or conditioned medium of MRL/*lpr* mice-derived BMCs (CM-MRL/lpr) (**B**) in the presence and absence of anti-mouse IL-17 antibody (Anti-IL17 Ab) or the control antibody (Cont Ab). (**C, D**) TRAP staining after co-culture of wild type mouse-derived BMCs (BMC-WT) and calvarial cells (Calvaria-WT) stimulated by 1α, 25(OH)$_2$ vitamin D$_3$ and prostaglandin E$_2$ under IL17 (**C**) or CM-MRL/lpr (**D**) in the presence and absence of Anti-IL17 Ab. n=5 for all groups. **P*<0.05, ****P*<0.005. The graph bars show means±SD.

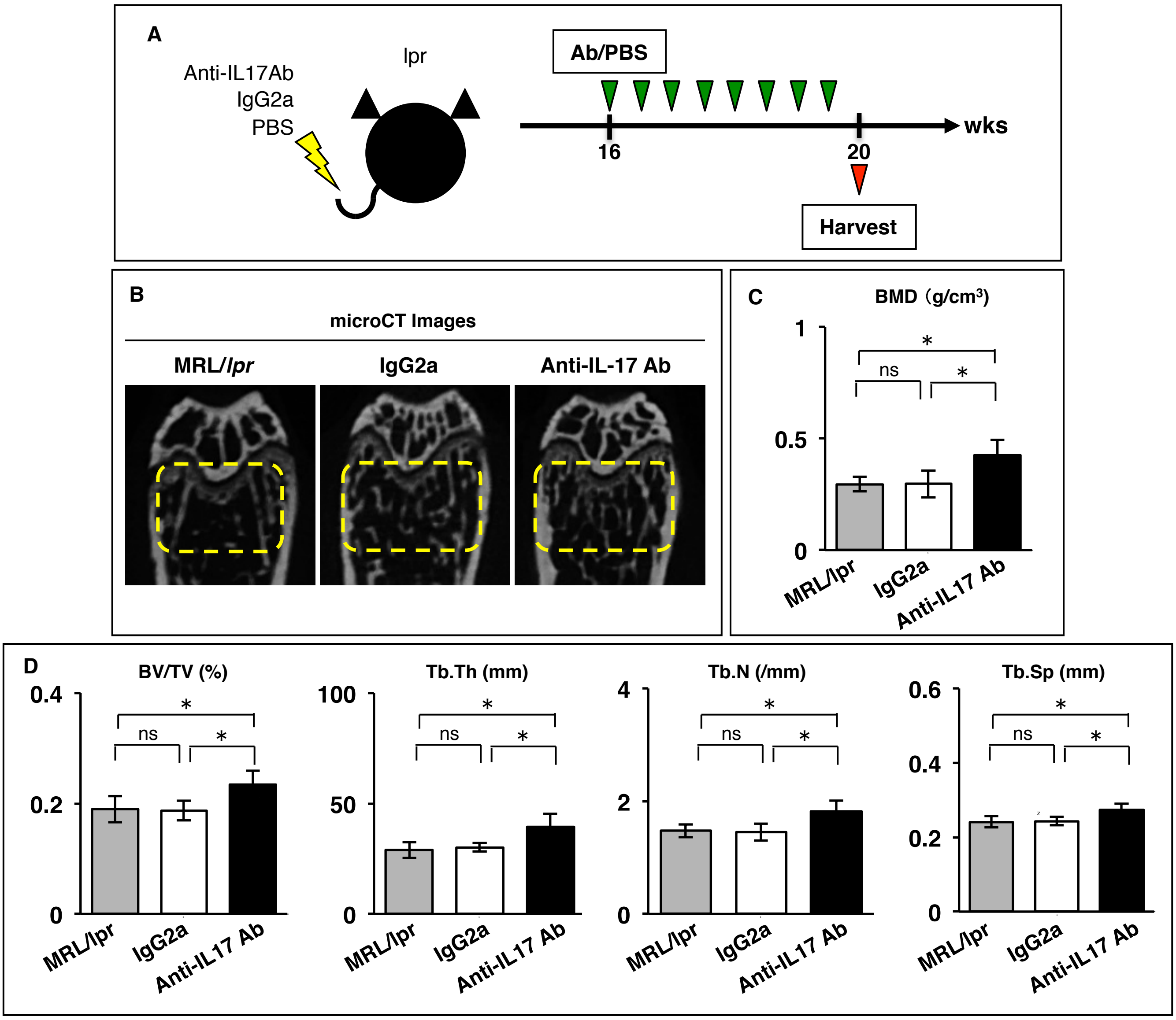

Figure S9. Systemic treatment of anti-IL-17 antibody improves bone loss in MRL/*lpr* mice. (**A**) A schema of systemic treatment of anti-IL-17 antibody (Anti-IL17 Ab) into MRL/*lpr* (lpr) mice. The antibody (1 mg/ml) was intraperitoneally injected twice a week for 4 weeks to MRL/*lpr* mice at 16 weeks (wks) of age. The control mice were treated with the isotype-matched IgG2a or PBS. **(B–D)** Morphological analyses of mouse tibiae. Representative microCT images of trabecular bone structures stage of MRL/*lpr* mice at 20 weeks of age (yellow-dashed areas) (**B**). Bone mineral density (BMD) (**C**). Trabecular bone parameter assay. BV/TV: bone volume ratio to tissue volume; Tb.Th: trabecular thickness; Tb.N: trabecular number; Tb.Sp: trabecular separation (**D**). **B-D:** n=5 for all group. MRL/lpr, IgG2a, Anti-IL17 Ab: non-, IgG2a and Anti-IL17 Ab-treated MRL/*lpr* mice at 20 weeks of age, respectively. *P<0.05. ns: no significance. The graph bars represent means±SD.

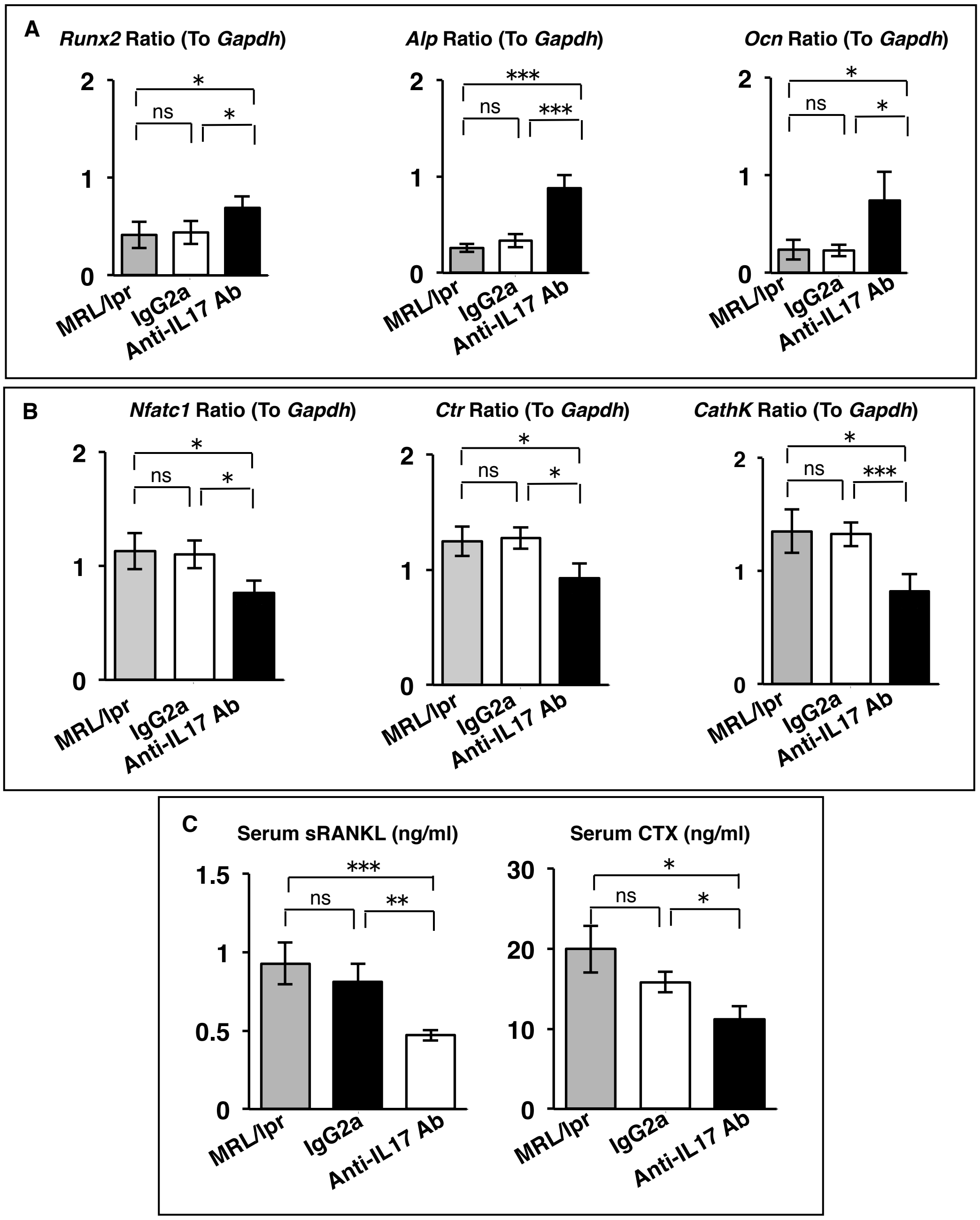

Figure S10. Systemic treatment of anti-IL-17 antibody regulates osteoblastogenesis and osteoclastogenesis via recipient BMMSCs in MRL/*lpr* mice. (**A**) *In vivo* osteoblast differentiation assay. Real-time RT-PCR analysis of osteoblast-specific genes in recipient long bones of MRL/*lpr* mice at 20 weeks of age. *Alp*: alkaline phosphatase; *Gapdh*: Glyceraldehyde 3-phosphate dehydrogenase; *Ocn*: osteocalcin; *Runx2*: runt-related transcription factor 2. (**B**) *In vivo* osteoclast differentiation assay. Real-time RT-PCR analysis of osteoclast-specific gene assay in recipient long bones of to MRL/*lpr* mice at 20 weeks of age. *Nfatc1*: nuclear factor of activated T cells, cytoplasmic 1; *Ctr*: calcitonin receptor, *CathK*: cathepsin K. (**C**) *In vivo* osteoclast activity assay. ELISA analyses of mouse serum. CTX: C-terminal telopeptides of type I collagen; sRANKL: soluble RANKL. **A-C:** n=5 for all groups. MRL/lpr, IgG2a, Anti-IL17 Ab: non-, IgG2a and Anti-IL17 Ab-treated MRL/*lpr* mice at 20 weeks of age, respectively. *$P<0.05$ and ***$P<0.005$. ns: no significance. Bar graphs show the means±SD.
